# Drinkable in situ-forming tough hydrogels for gastrointestinal therapeutics

**DOI:** 10.1038/s41563-024-01811-5

**Published:** 2024-02-27

**Authors:** Gary W. Liu, Matthew J. Pickett, Johannes L. P. Kuosmanen, Keiko Ishida, Wiam A. M. Madani, Georgia N. White, Joshua Jenkins, Sanghyun Park, Vivian R. Feig, Miguel Jimenez, Christina Karavasili, Nikhil B. Lal, Matt Murphy, Aaron Lopes, Joshua Morimoto, Nina Fitzgerald, Jaime H. Cheah, Christian K. Soule, Niora Fabian, Alison Hayward, Robert Langer, Giovanni Traverso

**Affiliations:** 1 David H. Koch Institute for Integrative Cancer Research, Massachusetts Institute of Technology, Cambridge, MA USA; 2 Department of Mechanical Engineering, Massachusetts Institute of Technology, Cambridge, MA USA; 3 Division of Gastroenterology, Hepatology, and Endoscopy, Brigham and Women’s Hospital, Harvard Medical School, Boston, MA USA; 4 Division of Comparative Medicine, Massachusetts Institute of Technology, Cambridge, MA USA; 5 Present Address: Department of Chemistry, Yale University, New Haven, CT USA; 6 Present Address: Fractyl Health, Inc., Lexington, MA USA; 7 Present Address: Weill Cornell Medical College, New York City, NY USA; 8 Present Address: Ross University School of Veterinary Medicine, Basseterre, St. Kitts and Nevis; 9 Present Address: Stanford University, Stanford, CA USA; 10 Present Address: Boston University, Boston, MA USA; 11 Present Address: MIT Media Lab, Cambridge, MA USA; 12 Present Address: Tufts University, Medford, MA USA; 13 Present Address: Broad Institute of MIT and Harvard, Cambridge, MA USA

**Keywords:** Drug delivery, Translational research, Biomedical engineering

## Abstract

Pills are a cornerstone of medicine but can be challenging to swallow. While liquid formulations are easier to ingest, they lack the capacity to localize therapeutics with excipients nor act as controlled release devices. Here we describe drug formulations based on liquid in situ-forming tough (LIFT) hydrogels that bridge the advantages of solid and liquid dosage forms. LIFT hydrogels form directly in the stomach through sequential ingestion of a crosslinker solution of calcium and dithiol crosslinkers, followed by a drug-containing polymer solution of alginate and four-arm poly(ethylene glycol)-maleimide. We show that LIFT hydrogels robustly form in the stomachs of live rats and pigs, and are mechanically tough, biocompatible and safely cleared after 24 h. LIFT hydrogels deliver a total drug dose comparable to unencapsulated drug in a controlled manner, and protect encapsulated therapeutic enzymes and bacteria from gastric acid-mediated deactivation. Overall, LIFT hydrogels may expand access to advanced therapeutics for patients with difficulty swallowing.

## Abstract (web summary)

Sequential drinks of crosslinker and polymer solutions form a tough hydrogel in the stomach, enabling delivery of drugs and biologics in this harsh chemical environment.

## Main

The oral route provides a safe, rapid and facile course for drug administration, and results in greater patient comfort and compliance compared with parenteral routes^1,2^. Due to advantages in stability, dose consistency and the capacity to co-formulate with excipients, oral solid drugs have become the predominant formulation: they consistently account for ∼50% of new US Food and Drug Administration (FDA)-approved drugs (https://www.fda.gov/), and nearly 70% of Americans are on at least one prescription drug^3^. However, certain patient populations struggle with swallowing solids. More than 50% of children are unable to swallow standard-sized pills or capsules^4^. Patients with dysphagia, or difficulty swallowing, similarly struggle with solid drug forms. In adults, the prevalence of dysphagia can be as a high as 16%, and upwards of 37% have difficulty swallowing pills^5,6^. This may cause patients to skip or modify (for example, crush) their medications, which may result in altered pharmacokinetic profiles and death^7^.

While liquid formulations are easier to ingest, they are susceptible to rapid dilution within the gastrointestinal tract and are unable to spatially localize drug with excipients^8^, which challenge efforts to orally deliver biological drugs. A system capable of a programmed liquid-to-solid transition within the stomach could bridge the advantages of these two forms. A solid matrix could facilitate spatial proximity of drug and excipients that modulate drug release or protect drug activity against the harsh gastric environment, and augment gastric residence of a drug depot. Efforts to develop liquid-to-solid systems have relied on drinkable hydrogel systems crosslinked by calcium. Orally administered calcium carbonate-loaded alginate solutions undergo gelation in the stomach due to acid-triggered release of Ca^2+^ ions and subsequent crosslinking of alginate^9^. Similarly, gellan or alginate solutions mixed with complexed calcium undergo in situ gastric gelation^10^. Oral administration of an alginate/karaya gum solution followed by a solution of CaCl_2_ results in gelation in the stomach^11^. An antiacid medication comprising alginate, sodium bicarbonate and calcium bicarbonate has also been described^12^. However, these single-network hydrogels are mechanically weak and may not be able to withstand compressive forces within the stomach (up to 13 kPa)^13^, resulting in irreversible deformation and potential breakage of the formulation.

**Fig. 1:**
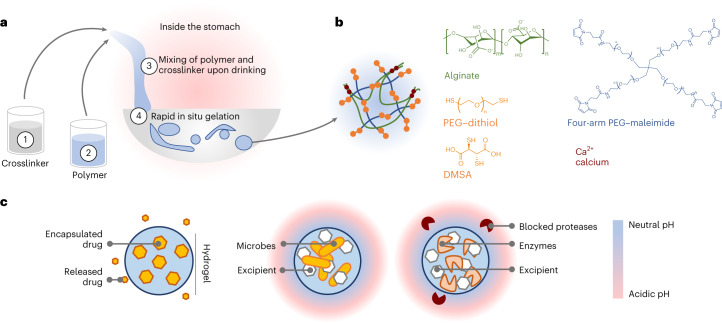
Overview of LIFT hydrogels. **a**, LIFT hydrogels form within the stomach after oral administration of (1) a 200-ml crosslinker solution comprising CaCl_2_ and a dithiol-containing molecule, followed by (2) a 20–40-ml polymer solution comprising alginate and four-arm PEG–maleimide. These two solutions (3) mix within the stomach to form a tough double-network hydrogel (4) within the stomach. **b**, Schematic of the polymers and reagents used to facilitate crosslinking. Materials were selected due to their established safety profiles. Both PEG–dithiol and dimercaptosuccinic acid (DMSA) were investigated as a dithiol crosslinker. **c**, Left: LIFT hydrogels may act as controlled release depots through encapsulation of water-insoluble drug that gradually dissolves and diffuses from the hydrogel. Middle and right: LIFT hydrogels enable co-encapsulation and co-localization of therapeutic microbes or enzymes and excipient (for example, CaCO_3_) that modulate local pH and protect against proteases.

In this Article, we describe a new strategy to enable a drinkable, liquid in situ-forming and tough (LIFT) hydrogel, which comprises both ionic (calcium/alginate) and covalent (poly(ethylene glycol) (PEG)) polymer networks for enhanced toughness^14^. LIFT hydrogels undergo gelation after the polymer solution containing alginate and functionalized PEG contacts the crosslinker solution within the stomach (Fig. 1a,b). We extensively characterize LIFT hydrogels after ex vivo formation in real gastric fluid and in vivo formation in rodent and large porcine models, and demonstrate that their capacity to form solids in situ enables these materials to act as a depot for controlled release of small molecules. Moreover, co-encapsulation with CaCO_3_ as an excipient protects the activity of orally delivered enzymes and therapeutic bacteria from the low pH of the stomach (Fig. 1c).

## Design and characterization of LIFT hydrogels

Due to the relatively short residence times (<30 min) of liquids in the stomach and the complexity of gastric fluid^15,16^, we sought to develop crosslinking chemistries that could rapidly and robustly crosslink two, interpenetrating polymer networks. Alginate is a well-studied, biocompatible polymer derived from algae with generally recognized as safe (GRAS) status that undergoes nearly instant crosslinking in the presence of calcium. We utilized PEG for the second network due to the established safety profiles of ingested PEGs^17^, and initially considered three crosslinking chemistries: *N*-hydroxysuccinimide (NHS) ester-amine, dibenzocyclooctyne (DBCO)-azide and maleimide-thiol. Due to the evolution of an uncharacterized and potentially toxic NHS leaving group during NHS ester-amine reaction and the slow kinetics ( > 1 h) of commercially available DBCO- and azide-functionalized PEGs (Supplementary Fig. 1), we proceeded with development of a PEG network crosslinked by maleimide-thiol reaction. Advantages of this chemistry include its rapid reaction kinetics, mild reaction conditions and biocompatibility^18^. To identify a safe dithiol crosslinker, we searched for FDA-approved or GRAS dithiol-containing small molecules for rapid diffusion and crosslinking of maleimide-functionalized PEG. Dimercaptosuccinic acid (DMSA) was selected due to its FDA approval status and well-characterized safety profile in humans^19,20^. A dithiol-terminated linear PEG (molecular weight 1,000 Da) was also selected for evaluation. Therefore, our final concept comprises (1) ingestion of a crosslinker solution comprising calcium chloride and DMSA or PEG–dithiol, followed by (2) ingestion of a polymer solution comprising alginate and four-arm PEG–maleimide. Upon (3) mixing in the stomach, the polymer solution undergoes crosslinking of both polymer networks and gelation to form (4) LIFT hydrogels (Fig. 1a,b).

**Fig. 2:**
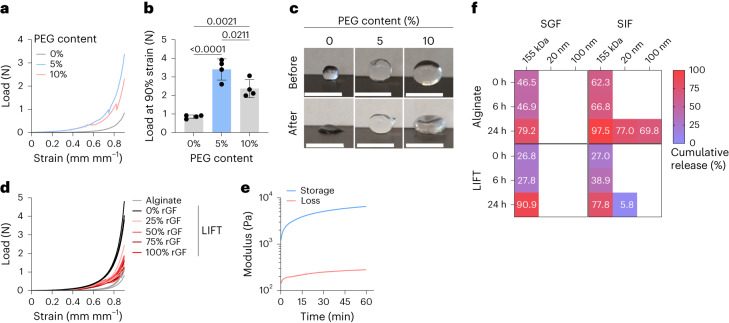
In vitro characterization of LIFT hydrogels. **a**, Representative load–strain curves of LIFT hydrogels comprising 0%, 5% or 10% w/v four-arm PEG–maleimide crosslinked in CaCl_2_/PEG–dithiol for 20 min, 37 °C, 50 RPM. **b**, Load at 90% strain of the different hydrogel compositions; *n* = 4 hydrogels were tested. Statistical analysis was performed by one-way ANOVA with post-hoc Tukey’s multiple comparisons test, *n* = 4 independent experiments. Data are presented as mean ± standard deviation. **c**, Images of various compositions of hydrogels before and after 90% strain. Scale bars, 5 mm. **d**, Load–strain curves of LIFT hydrogels formed in various % v/v mixtures of real porcine gastric fluid (rGF) and water containing CaCl_2_/PEG–dithiol. **e**, Gelation kinetics of LIFT hydrogels immersed in a crosslinker bath comprising CaCl_2_/PEG–dithiol at 37 °C, as characterized by rheology. **f**, Cumulative release of 155 kDa dextran and 20- or 100-nm nanoparticles from alginate and LIFT hydrogels. Hydrogels were incubated in SGF or SIF for the indicated time periods. Shown is the average of *n* = 3 independent experiments. Source data

We first asked whether LIFT hydrogels were capable of forming under short (20 min) timeframes relevant to gastric residence of ingested liquids. To emulate in vivo formation conditions, a 0.5% w/v solution of alginate with 0%, 5% and 10% w/v four-arm PEG–maleimide was drop cast into a crosslinker solution (200 mM CaCl_2_/10 mM PEG–dithiol or DMSA) and then incubated for 10–20 min at 37 °C. The resulting hydrogels were mechanically characterized by compression testing. LIFT hydrogels sustained significantly greater loads compared with alginate hydrogels (Fig. 2a,b and Supplementary Fig. 2a). After 90% strain, LIFT hydrogels remained mostly spherical, whereas alginate hydrogels remained permanently deformed (Fig. 2c and Supplementary Fig. 2b). LIFT hydrogels were further mechanically characterized by cyclic compression testing. While LIFT hydrogels could sustain at least five cycles of 90% strain, alginate hydrogels remained permanently deformed after one cycle and were unable to sustain subsequent strains (Supplementary Fig. 3). Due to the greater mechanical performance and easier manipulation of 5% w/v PEG-containing LIFT hydrogels compared with 10% w/v PEG, this composition was further characterized. This observation may be due to greater dissolution and mixing of the 5% w/v PEG with alginate. To test the capacity of LIFT hydrogels to form in vivo, hydrogels were formed in fresh porcine gastric fluid at various dilutions in water. As a control, hydrogels were compared with LIFT or alginate hydrogels formed in the absence of gastric fluid. While gastric fluid attenuated the mechanical properties of LIFT hydrogels, these hydrogels were mechanically tougher than alginate hydrogels formed under ideal conditions (Fig. 2d and Supplementary Fig. 2c). LIFT hydrogel components were also tested for cytotoxicity in a variety of cell lines. After 24 h of continuous incubation at relevant concentrations, no major causes of cytotoxicity were observed (Supplementary Fig. 4). Collectively, these data demonstrate that LIFT hydrogels can form rapidly even in gastric fluid, the resulting hydrogels are mechanically tough, both DMSA and PEG–dithiol crosslinkers are capable of crosslinking the covalent PEG network, and the hydrogel components are biocompatible.

The kinetics of LIFT hydrogel formation were further studied by rheometry. To emulate rapid alginate crosslinking and to facilitate experimentation, hydrogels were first internally crosslinked with CaCO_3_ and glucono-δ-lactone and then analysed within a bath of 200 mM CaCl_2_/10 mM PEG–dithiol solution. Notably, the sharpest increase in modulus occurred during the first 10–15 min (Fig. 2e), further supporting the feasibility of gastric crosslinking at timeframes relevant to liquid retention within the stomach (time of 50% emptying: 15–30 min)^15,21^. LIFT hydrogels were then studied for their capacity to encapsulate therapeutic cargos of different length scales, using 155-kDa dextran as a model macromolecule and 20- or 100-nm polystyrene nanoparticles as model controlled-release nanoparticles. LIFT or alginate hydrogels encapsulating these model cargoes were immersed in simulated gastric fluid (SGF, pH 1.77) or simulated intestinal fluid (SIF, pH 6.8), which were sampled at various timepoints. Neither hydrogels were able to detain dextran in either medium (>75% release); however, LIFT hydrogels exhibited less nanoparticle release in SIF (<1–6%) compared with alginate hydrogels after 24 h (70–77%, Fig. 2f). This is consistent with prior reports describing the increased pore sizes and release of alginate hydrogels in alkaline environments^22,23^. Therefore, LIFT hydrogels may be capable of retaining therapeutic cargoes at a variety of length scales due to greater stability at various pH ranges and/or smaller pore sizes.

## Characterization of LIFT hydrogels in large animals

**Fig. 3:**
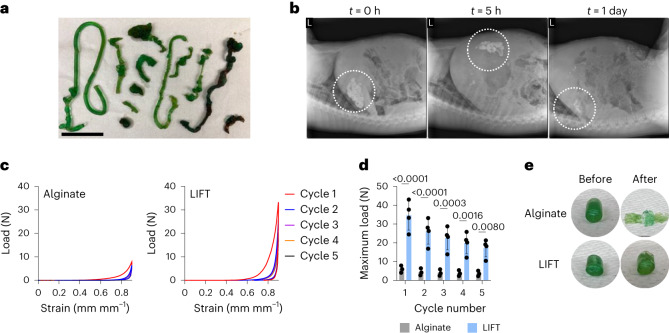
In vivo characterization of LIFT hydrogels. **a**, Hydrogel geometries after in vivo formation in female Yorkshire pigs. LIFT hydrogels were formed by endoscopic administration of crosslinker solution (200 mM CaCl_2_/10 mM PEG–dithiol) followed by polymer solution (0.5% alginate/5% w/v four-arm PEG–maleimide). Scale bar, 5 cm. **b**, X-ray imaging of LIFT hydrogels in female Yorkshire pigs throughout time. Shown is representative of *n* = 3 independent pig experiments. **c**, Load–strain curves of alginate or LIFT hydrogels after retrieval from Yorkshire pig stomachs. Hydrogels were characterized by five cycles of 90% strain. **d**, Maximum loads experienced by alginate or LIFT hydrogels throughout five cycles of 90% strain. Statistical analysis was performed by two-way ANOVA with post-hoc Šidák’s multiple comparisons test, *n* = 3 (alginate) or 4 (LIFT) independent experiments. Data are presented as mean ± standard deviation. **e**, Images of retrieved alginate or LIFT hydrogels before and after 90% strain. Source data

LIFT hydrogels were then tested for formation, kinetics and safety in vivo. Porcine models were used due to the similarity of their gastrointestinal tract size to that of humans. First, the administration order of crosslinker (200 mM CaCl_2_/10 mM DMSA or PEG–dithiol) and polymer solutions (0.5% alginate/5% w/v four-arm PEG–maleimide) was varied. Pigs were administered solutions into the stomach via endoscope, and hydrogel structures were observed 5–8 h afterwards. Hydrogels formed within the stomach cavity regardless of administration order. Administration of crosslinker first and then polymer solution resulted in the reproducible formation of noodle-like hydrogels within the stomach; the reverse order resulted in larger, but less consistent, bulk hydrogels (Fig. 3a and Supplementary Fig. 5). Accordingly, LIFT hydrogels were formed in vivo by first administration of the crosslinker followed by the polymer solution. LIFT hydrogels were then studied for their transit time in vivo through X-ray imaging of hydrogels containing 20% w/v barium sulfate. In general, LIFT hydrogels remained within the stomach up to 24 h after administration (Fig. 3b and Supplementary Fig. 6); in comparison, liquids are emptied from the porcine stomach in 0.4–1.4 h across fasted and fed states^24^. No changes in liver or renal function were observed up to 48 h after administration (Supplementary Table 1). These data support that LIFT hydrogels and their components are safely cleared, do not cause obstruction and do not cause toxicity. After formation in the gastric cavity, LIFT hydrogels were characterized for their mechanical properties by cyclic compression testing. LIFT hydrogels were tougher and able to sustain at least five cyclic 90% strains, whereas alginate hydrogels remained flattened after one cycle (Fig. 3c–e and Supplementary Fig. 7). Moreover, in ex vivo tissue experiments, we did not observe differences in hydrogel yield (in terms of mass) in complex environments or evidence of hydrogel adhesion to tissue (Supplementary Fig. 8 and Supplementary Discussion). These findings highlight the capacity of the LIFT hydrogels to robustly form in the stomach after oral administration in a human-scale gastrointestinal tract.

## LIFT hydrogels modulate small molecule release

**Fig. 4:**
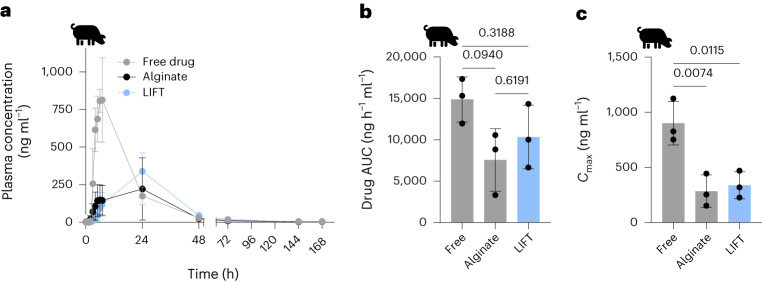
Pharmacokinetics of various oral lumefantrine formulations. **a**, Plasma lumefantrine concentration over time of free lumefantrine and lumefantrine encapsulated in alginate or LIFT hydrogel. For each treatment, *n* = 3 female Yorkshire pigs were tested. **b**, Lumefantrine AUC of each formulation. **c**, Maximum observed lumefantrine concentration (*C*_max_) of each formulation. For **b** and **c**, statistical analysis was performed by one-way ANOVA with post-hoc Tukey’s multiple comparisons test, *n* = 3 Yorkshire pigs per treatment. All data are presented as mean ± standard deviation. Source data

Having established that LIFT hydrogels can form in vivo, we evaluated their capacity to encapsulate and modulate small molecule release. We selected lumefantrine as a model small molecule drug because it has poor solubility in water and, thus, would form a drug suspension that is encapsulated within the hydrogel after formation. Hydrogels were administered into the stomach of pigs using 200 mM CaCl_2_/10 mM DMSA as the crosslinker solution; lumefantrine was suspended in 0.5% alginate/5% w/v four-arm PEG–maleimide LIFT polymer solution. Lumefantrine powder loaded in gelatin pills was used as a free drug control, and all pigs were dosed with 960 mg lumefantrine. Whereas free lumefantrine resulted in peak plasma concentrations at 5–7 h post-administration, hydrogel (alginate and LIFT) formulations resulted in peak plasma drug concentrations at ∼24 h (Fig. 4a). The area under the curve (AUC) of released drug from free drug, alginate and LIFT hydrogel was 14,873.5 ± 2,719.2, 7,568.4 ± 3,780.6 and 10,337.5 ± 3,849.7 ng h^−1^ ml^−1^, respectively, and was not statistically different (Fig. 4b). While drug AUCs did not differ, the maximum observed drug concentration (*C*_max_) was significantly higher with free drug (901.2 ± 197.1 ng ml^−1^) compared with alginate (283.8 ± 147.3 ng ml^−1^) and LIFT (338.7 ± 122.6 ng ml^−1^) hydrogel (Fig. 4c). These data collectively support the capacity of LIFT hydrogels to deliver comparable total doses of drug as free drug at lower plasma concentrations, which may reduce drug toxicity^6,25^.

## LIFT hydrogels protect therapeutic enzyme activity

**Fig. 5:**
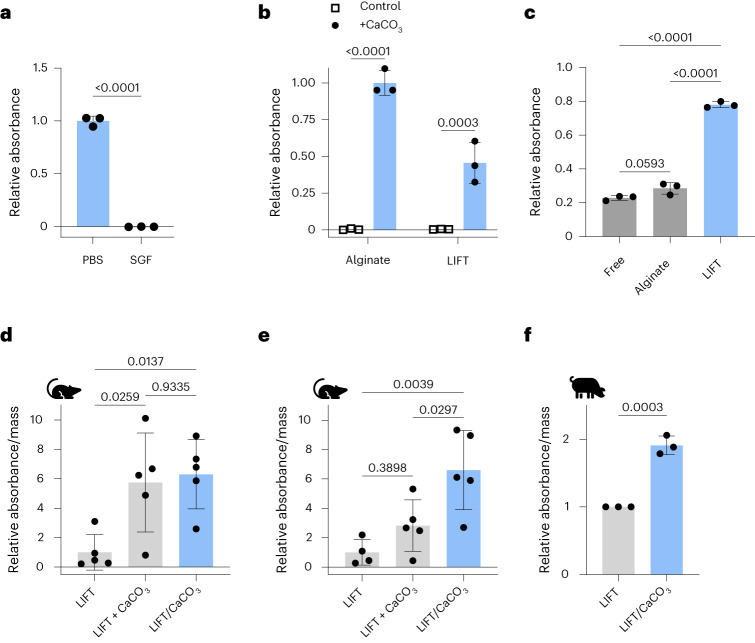
LIFT hydrogel co-encapsulation of CaCO_3_ protects lactase activity after oral delivery. **a**, Lactase activity, as measured by ONPG assay, after 15 min incubation in PBS or SGF at 37 °C. Absorbances were normalized to that of lactase incubated in PBS. Statistical analysis was performed by two-tailed Student’s *t*-test, *n* = 3 independent experiments. **b**, Lactase activity after hydrogel encapsulation with or without CaCO_3_ co-encapsulation and incubation in SGF for 1 h. Absorbances were normalized to that of alginate/CaCO_3_. Statistical analysis was performed by two-way ANOVA with post-hoc Šidák’s multiple comparisons test, *n* = 3 independent experiments. **c**, Lactase activity of various treatments after trypsin treatment. Absorbances were normalized to that of treatment without trypsin. Statistical analysis was performed by one-way ANOVA with post-hoc Tukey’s multiple comparisons test, *n* = 3 independent experiments. **d**, Activity of lactase encapsulated in LIFT hydrogels after 1 h in male Sprague–Dawley rats. CaCO_3_ was administered separately (LIFT + CaCO_3_) or co-encapsulated (LIFT/CaCO_3_). Absorbances were normalized by hydrogel mass. Statistical analysis was performed by one-way ANOVA with post-hoc Tukey’s multiple comparisons test, *n* = 5 rats per treatment. **e**, Activity of lactase encapsulated in LIFT hydrogels after 2 h in male Sprague–Dawley rats. Absorbances were normalized by hydrogel mass. Statistical analysis was performed by one-way ANOVA with post-hoc Tukey’s multiple comparisons test, *n* = 4 (LIFT) or 5 rats (LIFT+CaCO_3_, LIFT/CaCO_3_). **f**, Activity of lactase encapsulated in LIFT hydrogels after 6 h in female Yorkshire pigs. Hydrogels were retrieved from porcine stomach and randomly sampled. Absorbances were normalized by hydrogel mass and to control hydrogels without CaCO_3_. Statistical analysis was performed by two-tailed Student’s *t*-test, *n* = 3 independent pig experiments. All data are presented as mean ± standard deviation. Source data

We next evaluated the capacity of LIFT hydrogels for oral delivery of enzymes, which is challenging due to the acidic gastric fluid and proteases present within the gastrointestinal tract^26^. β-Galactosidase (lactase) was selected due to the need for prolonged lactase activity in the stomach to mitigate the symptoms of lactose intolerance^27^. Indeed, lactase activity was significantly reduced when incubated in SGF compared with phosphate-buffered saline (PBS; Fig. 5a). Lactase was then encapsulated in alginate or LIFT hydrogels along with CaCO_3_ as an excipient to neutralize the acidic gastric fluid. CaCO_3_ was selected because it is water insoluble and therefore detainable within hydrogels, and because of its GRAS status. Because the DMSA crosslinker attenuated lactase activity (Supplementary Fig. 9), these LIFT hydrogels utilized a PEG–dithiol crosslinker. When challenged with SGF, CaCO_3_-containing hydrogels preserved lactase activity (Fig. 5b), underscoring the compatibility of LIFT hydrogels with enzymes. In addition to acidic gastric fluid, the gastrointestinal tract is rife with proteases that can degrade enzymes. We asked if the hydrogels were capable of protecting against trypsin as a model protease. LIFT hydrogels exhibited the greatest protection of lactase activity compared with free lactase or lactase encapsulated in alginate hydrogels (Fig. 5c). Therefore, in addition to co-encapsulating CaCO_3_, LIFT hydrogels may exhibit additional barriers against proteases due to the denser, dual polymer networks compared with alginate hydrogels^14^.

LIFT hydrogels were then tested for their ability to protect lactase activity in vivo. Similar to studies performed in pigs, rats were first administered crosslinker solution by oral gavage immediately followed by polymer solution containing lactase with or without CaCO_3_; as an additional control, CaCO_3_ was suspended in the crosslinker solution. Each animal was treated with a CaCO_3_ dose less than the maximum daily dose of 7–10 g per day (assuming a 75 kg human) established by manufacturers^28^. Therefore, these set of treatments test the effect of CaCO_3_ administered separately (LIFT + CaCO_3_) or co-encapsulated (LIFT/CaCO_3_). Oral gavage resulted in robust hydrogel formation in rat stomachs (Supplementary Fig. 10), and hydrogels were retrieved after in vivo incubation in stomachs and assayed for lactase activity. Notably, while separate and co-encapsulated CaCO_3_ significantly protected lactase activity after 1 h (Fig. 5d), only co-encapsulated CaCO_3_ protected lactase after 2 h (Fig. 5e). The protective effect of the LIFT hydrogels co-encapsulating CaCO_3_ was also observed in porcine models after a 6 h in vivo incubation (Fig. 5f), underscoring the advantage of oral systems capable of excipient co-encapsulation in large animal models. We further validated the broader compatibility of LIFT with additional enzymes in vitro and in vivo in rats (Supplementary Fig. 11 and Supplementary Discussion).

## LIFT hydrogels protect therapeutic bacteria viability

**Fig. 6:**
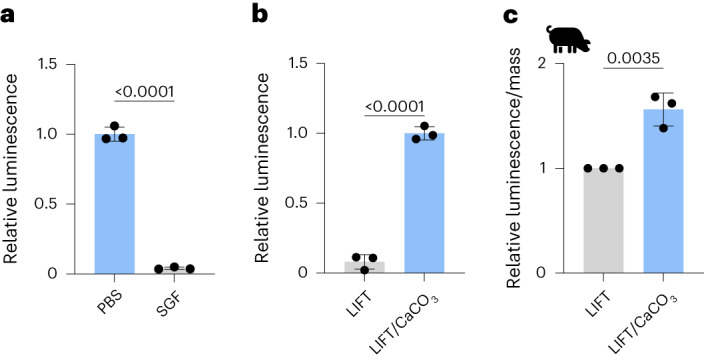
LIFT hydrogel co-encapsulation of CaCO_3_ protects bacterial activity. **a**, *L. lactis* viability, as measured by a luminescent ATP quantification assay, after 10 min incubation in PBS or SGF at 37 °C. Luminescence was normalized to that of bacteria incubated in PBS. Statistical analysis was performed by two-tailed Student’s *t*-test, *n* = 3 independent experiments. **b**, Viability of *L. lactis* encapsulated in LIFT hydrogels with or without CaCO_3_ and incubated in SGF for 3 h. Statistical analysis was performed by two-tailed Student’s *t*-test, *n* = 3 independent experiments. **c**, Viability of *L. lactis* encapsulated in LIFT hydrogels after 6–7 h in female Yorkshire pigs. Hydrogels were retrieved from porcine stomach and randomly sampled. Luminescence values were normalized by hydrogel mass and to control hydrogels without CaCO_3_. Statistical analysis was performed by two-tailed Student’s *t*-test, *n* = 3 independent pig experiments. All data are presented as mean ± standard deviation. Source data

Another class of biologics of interest for oral delivery are therapeutic bacteria such as *Lactococcus* *lactis*, which is a critical chassis for synthetic biology therapeutics^29,30^. Oral delivery of bacteria is a recognized challenge and currently requires large solutions of sodium bicarbonate to buffer stomach pH^31^. We utilized a luminescent ATP quantification assay to query bacterial viability. *L. lactis* exhibited decreased viability after short exposure to SGF pH 1.77 (Fig. 6a), which was mitigated when co-encapsulated with CaCO_3_ within LIFT (Fig. 6b). At 6–7 h post-administration within porcine stomachs, *L. lactis* co-encapsulated with CaCO_3_ within LIFT exhibited greater viability compared with control (Fig. 6c). Similar experiments were performed with *Escherichia* *coli* Nissle 1917 (EcN) but did not exhibit statistical significance (Supplementary Fig. 12 and Supplementary Discussion). The differences in response between EcN and *L. lactis* may be due to greater susceptibility of the latter to pH ranges relevant to gastric acid^32^. Thus, LIFT hydrogels are capable of supporting bacterial viability and protect against acid challenge when loaded with CaCO_3_ in an in vivo context. Given that acid secretion can vary 40–71 mmol h^−1^ (interquartile range) in humans and the recommended maximum recommended dose of CaCO_3_ (10 g per day)^28,33^, these systems could potentially support the viability of bacteria in the stomach for 3–5 h.

## Outlook

Dysphagia and difficulty swallowing present major obstacles to oral drug administration. Here we developed a drinkable formulation, called LIFT hydrogels, capable of transitioning from liquid to solid upon mixing in the stomach. LIFT hydrogels possess advantages of solid formulations, including enhanced gastric retention, protection against gastrointestinal proteases, mechanical toughness, capacity to control drug release, and co-encapsulation of sensitive therapeutics with excipients. To realize LIFT hydrogels, we used FDA-approved or GRAS materials: alginate and four-arm PEG–maleimide as hydrogel networks, and calcium chloride and DMSA or PEG–dithiol as crosslinkers. The polymer solution remains a liquid until contact with the crosslinker within the stomach, triggering a transition from a liquid to a tough hydrogel.

Gastric drug depots should be able to withstand the compressive forces of the gastrointestinal tract to preserve depot integrity. While double-network hydrogels are mechanically tough^14^, current strategies to formulate such hydrogels from orally administered liquids have not been described. While we and others have developed orally administrable tough hydrogels^34,35^, these require templated radical polymerization of toxic acrylamide that cannot be safely performed in vivo^36^. Li et al. utilized pH-triggered unmasking of multivalent cyclodextrin to undergo gelation with multivalent adamantane in acidic conditions^37^; however, the liberated masking group will need to be characterized for safety. Other hydrogel systems require ultraviolet light to facilitate crosslinking^38^, utilize polyacrylamide^14^, require a specific construction of hydrogel components^34^, or are enzymatically polymerized^39^. However, these systems require a solid dosage format or are challenging and unsafe to crosslink in situ. This work bridges this gap and enables liquid formulation of a tough hydrogel. Assuming a spherical hydrogel, we calculate that reported gastric stresses (∼13 kPa) would cause a strain of 5–10% in LIFT hydrogels, which should not permanently deform these materials^13^.

The gastric environment exhibits some features amenable for in situ crosslinking. The stomach is temperature controlled at 37 °C, which can accelerate maleimide-thiol thioether formation^40^; the stomach is also mechanically active which could facilitate mixing of the ingested solutions^13^. We demonstrate that crosslinking of both polymer networks readily occurs in ex vivo porcine gastric fluid and in vivo in porcine and rat stomachs, which underscores the robustness of the crosslinking chemistry. While maleimide-thiol reactions are rapid^18^, inclusion of alginate augments the mechanical properties of the LIFT hydrogel. Moreover, its immediate crosslinking in calcium acts as a ‘scaffold’ that facilitates retention and crosslinking of the slower-forming thioether bond. Highly defined maleimide:thiol ratios are typically required for efficient crosslinking that is challenging to implement in an oral setting^18,40^; here, scaffolding within alginate probably enables gradual diffusion of the dithiol into the hydrogel and crosslinking of the PEG network. While LIFT hydrogel formation and mechanical properties were dependent on the proportion of gastric fluid volume, this may be diluted through greater volumes of crosslinker. The fasted stomach contains 25–35 ml of gastric fluid^21,41^, which after ingestion of a 200 ml crosslinker solution is diluted to 11–15%. This volume is less than the volume of a typical drink can (355 ml), and this proportion of gastric fluid is well within the range capable of crosslinking LIFT. Notably, these reactions do not generate side products, and the hydrogels did not appear to be toxic to cultured cells nor cause clinical (for example, constipation and inappetence) or blood chemistry signals in pigs up to 48 h after administration.

An important advantage that LIFT confers over particulate suspensions is the in situ gelation of macroscale structures, which is important to minimize exposure of the dosage forms to gastric fluid. Given the same volume of material, nano- and microscale dosage forms result in greater surface area-to-volume ratios compared with macroscale forms. Economou et al. showed that the dissolution rate of CaCO_3_ particles was size dependent, with larger 2–4 mm particles dissolving slower than <250 µm particles in acid^42^. Therefore, in situ gelation of macrostructures could enable protection of encapsulated therapeutics through size and geometry. Moreover, the formation of macroscale solids could prolong the gastric retention of encapsulated drugs compared with particulate suspensions^15^.

We show that LIFT hydrogels modify pharmacokinetics by delaying and reducing the maximum drug plasma concentration while achieving a comparable drug AUC as free drug. This is important because high drug concentrations can result in adverse side effects and impact treatment tolerability^43^, and is applicable for drugs in which efficacy is driven by AUC and not blood concentration (for example, tetracyclines)^44^. LIFT could also control water-soluble drug release, which would require particle encapsulation or covalent attachment to LIFT polymers to prevent burst release. Formulation in LIFT confers additional advantages of longer transit times and reduced surface area-to-volume ratios that could further control release; moreover, inclusion of protease- or pH-sensitive linkers could enable programmed release within specified tissues.

As a range of molecules are in equilibrium between the blood and gastrointestinal tract^45^, oral therapeutics that modulate these molecules within the stomach could noninvasively treat disease. As examples, engineered bacteria can sense and metabolize specified molecules, and enzyme therapies are being developed for the treatment of hyperoxaluria and phenylketonuria^46–49^. Coupling LIFT hydrogels with these therapeutics could prolong their residence and activity in a tough form factor. We envision that LIFT could serve as a compliant ‘niche’ by co-encapsulating excipients (for example, CaCO_3_) that modulate the local environment and the therapeutic themselves. As LIFT hydrogels transit through the gastrointestinal tract, the porosity of the hydrogels facilitates access to host metabolites and secretion of therapeutic factors.

By overcoming the ‘ship-in-a-bottle’ problem, LIFT hydrogels could expand access to advanced therapeutics for patients who have difficulty swallowing solids and bridge the advantages of solid and liquid drug formulations. We envision that LIFT hydrogels and their flexible chemistries may be a useful strategy with wide applications in gastric drug modulation and delivery, weight loss and protection of encapsulated biologics.

## Methods

### Chemicals

PEG–dithiol (1 kDa) was purchased from BioPharma PEG, four-arm PEG–maleimide (20 kDa) was purchased from JenKem Technology USA, Laysan Bio and Creative PEGWorks, and alginate (71238), trypsin (T7409), α-galactosidase (G8507), cellulase (C1794) and β-galactosidase (G5160) were purchased from MilliporeSigma. Alginate solutions were prepared in ddH_2_O by vigorous heating and stirring. Calcium carbonate and DMSA were purchased from ACROS Organics, and *o*-nitrophenyl β-d-galactopyranoside (ONPG) and X-α-Gal were purchased from Cayman Chemical. Lumefantrine and EnzChek Cellulase Substrate were purchased from Fisher Scientific, and halofantrine was purchased from MedChemExpress.

### In vitro LIFT hydrogel formation and characterization

A polymer solution of 0.5% w/v alginate and four-arm PEG–maleimide (0-10% w/v) was prepared in ddH_2_O; to form hydrogels, 60 μl of this solution was cast into 1 ml of crosslinker solution (200 mM CaCl_2_, 10 mM PEG–dithiol or DMSA) using a pipette and incubated for 10–20 min at 37 °C, 50 RPM. Then, the resulting hydrogels were washed with ddH_2_O. In some experiments, hydrogels were cast in 0–100% v/v of porcine gastric fluid diluted with ddH_2_O. Concentrated stocks of crosslinker were added to porcine gastric fluid to achieve the stated crosslinker concentrations and % v/v gastric fluid.

Mechanical compression tests were performed using an Instron instrument. The gauge length was determined with a digital caliper, and displacement was applied at a rate of 0.05 mm s^−1^ until 90% strain. Cyclic compression measurement was performed with a displacement rate of 0.05 mm s^−1^ and five cycles of 90% strain.

To facilitate rheological characterization, pre-crosslinked LIFT hydrogels were prepared by casting a solution of 0.5% alginate/5% w/v four-arm PEG–maleimide with 15 mM CaCO_3_/30 mM glucono-δ-lactone into a 100-mm Petri dish. After 1 h incubation at room temperature, hydrogel samples were made using an 8-mm-diameter biopsy punch. Oscillatory rheology studies were performed with a Discovery Series Hybrid Rheometer from TA Instruments. Samples were measured using 8-mm parallel plates fully submerged in a 5-ml bath of crosslinker solution (200 mM CaCl_2_/10 mM PEG–dithiol) at 37 °C. We reasoned that the 8-mm parallel plates (smallest available size) would minimize unexposed surface area at the top and bottom faces of the sample, and therefore best represent crosslinking dynamics in vivo. Data were collected for 1 h with a frequency of 10 rad s^−1^ and strain of 1%.

### Model therapeutic encapsulation and release

The following model encapsulants were mixed at a 10 mg ml^−1^ concentration in either alginate or LIFT polymer solutions: 155-kDa tetramethylrhodamine isothiocyanate-dextran (MilliporeSigma), and 20- and 100-nm fluorescent carboxylated polystyrene nanoparticles (ThermoFisher). Hydrogels were formed as described above, transferred to SGF (34 mM NaCl pH 1.77) or SIF (Cole-Parmer), and then incubated at 37 °C, 50 RPM. The supernatant was sampled at various timepoints with replacement. For each hydrogel and model encapsulant, three separate experiments were performed simultaneously, and release was calculated according to respective standards prepared in SGF or SIF.

### Cytotoxicity

Cytotoxicity of gel constituents (four-arm PEG–maleimide, PEG–dithiol, DMSA and CaCl_2_) was determined for four different cell lines: Caco-2, HT-29, Hepa1-6 and CV-1 (all obtained from American Type Culture Collection). Additional information regarding cell lines is provided in Supplementary Table 2. Cell lines were mycoplasma tested as negative before use, and genomically fingerprinted, where possible, to verify their identity. Alginate was unable to be tested due to its viscosity and incompatibility with robotic fluid handlers. Cells were plated at 15,000 cells per well in Dulbecco’s modified Eagle medium + 10% foetal bovine serum using robotic handlers (Tecan Evo 150) and incubated overnight. Then, cells were incubated in the indicated treatments and concentrations for 24 h in Dulbecco’s modified Eagle medium + 10% foetal bovine serum, and viability was quantified using CellTiter-Glo (Promega), which uses intracellular ATP levels as a surrogate for viability, and a plate reader (Tecan Infinite Pro 1000). Viability was calculated as a percentage of untreated cells.

### In vivo experimentation

All animal studies were performed only after Massachusetts Institute of Technology Committee on Animal Care review and approval and under veterinary supervision (protocol numbers 2203000114 and 2207000395). The Massachusetts Institute of Technology Division of Comparative Medicine provided guidance and training. Specific methods and treatments for characterization, lumefantrine, lactase and bacteria studies in rats and pigs are described within their respective sections. Female Yorkshire pigs aged 3–7 months (50–100 kg, sourced from Animal Biotech Industries (Doylestown, PA) or Tufts University Cummings School of Veterinary Medicine (Grafton, MA)) and male or female Sprague–Dawley rats (>400 g, sourced from Charles River Laboratories (Wilmington, MA), strain code 001) were used. Due to limited supplies of large >400 g rats, rats were used regardless of age. No animals were excluded from analysis. Animals were randomized to treatment groups.

### In vivo LIFT hydrogel formation and characterization

Hydrogels were administered into stomachs of anaesthetized pigs via endoscopy. To facilitate visualization, gastric fluid was removed. Crosslinker solution (200 ml, 200 mM CaCl_2_ and 10 mM DMSA or PEG–dithiol) was first administered, and then the endoscope was purged with air and water. After, 20–40 ml of polymer solution (typically 0.5% alginate/5% w/v four-arm PEG–maleimide) was similarly administered. In some experiments, the order was reversed. For mechanical characterization, pigs were euthanized 6–8 h after hydrogel administration, and the hydrogels were retrieved and tested as described above.

To monitor hydrogel retention kinetics in the porcine gastrointestinal tract and for acute toxicity, hydrogels were loaded with barium sulfate (20% w/v) for X-ray imaging, and images were collected immediately after administration, 4–5 h, and on days 1 and 2. Serum was collected before hydrogel administration (baseline) and on days 1 and 2 for metabolic analysis. Throughout, pigs were clinically monitored for gastrointestinal symptoms (for example, inappetence and vomiting).

### Ex vivo LIFT characterization

LIFT hydrogels were characterized for yield and mechanical properties after formation in a gastric tissue environment or normal plastic plate as a control. To recreate an ex vivo gastric tissue environment, abattoir-sourced porcine stomachs were cut into strips and briefly washed with ddH_2_O. Tissue was then applied to a plate and secured with a magnetic device that creates individual wells for experimentation. Crosslinker solution (400 μl, 200 mM CaCl_2_/10 mM DMSA or PEG–dithiol) was applied to these wells or the wells of a 48-well plate, and then 50 μl of polymer solution (0.5% alginate/5% w/v four-arm PEG–maleimide) was drop cast into these wells. After incubation for 20 min at 37 °C, 50 RPM, hydrogels were briefly washed with ddH_2_O and then weighed. These same hydrogels were mechanically characterized as described above.

To test LIFT hydrogel adhesion to gastric tissue, hydrogels were applied to the centre of each well of magnetic device-secured gastric tissue and then incubated for 5 min at 37 °C. Then, the plate was tilted at ∼45°, and 400 μl ddH_2_O was added and then removed from each well. Hydrogel location within the well was recorded before and after washing and tilting.

### Encapsulated lumefantrine pharmacokinetics

Pigs were dosed under anaesthesia via endoscopy with the following treatments: free lumefantrine, lumefantrine encapsulated in alginate hydrogels, and lumefantrine encapsulated in LIFT hydrogels (*n* = 3 each). All pigs were dosed with a total of 960 mg lumefantrine. For free lumefantrine, drug powder was weighed and placed across three gelatin capsules. For hydrogel formulations, lumefantrine powder was suspended in polymer solution (0.5% w/v alginate or 0.5% alginate/5% w/v four-arm PEG–maleimide), mixed, and administered after crosslinker solution (200 mM CaCl_2_/10 mM DMSA). Blood was sampled from a central jugular catheter at the indicated timepoints, and lumefantrine AUC was calculated by the trapezoidal rule.

Plasma lumefantrine was separated via high-performance liquid chromatography and quantified with an Agilent 6495A triple quadrupole mass spectrometer equipped with a sheath gas electrospray ionization (Agilent Technologies). Samples were injected at a 5 μl injection volume. Chromatography was performed on an Acquity BEH C18 column (2.1 × 50 mm, (particle diameter) *d*_p_ = 1.8 μm, Waters), heated to 50 °C, with a binary mobile phase composed of 0.1% formic acid in water (v/v, A) and 5% tetrahydrofuran in methanol (v/v, B). The mobile phase was pumped at 0.5 ml min^−1^ and gradient programmed as follows: 0 min, 5% B; 5 min, 95% B. The total method runtime was 7 min with a 2-min re-equilibration time between injections. For positive ionization electrospray ionization source conditions, the iFunnel high pressure radiofrequency was set to 150 V, and low pressure set to 60 V. Nebulizer drying gas temperature was set to 210 °C with a flow rate of 15 l min^−1^ at 35 psig. Sheath gas temperature was set to 250 °C with a flow rate of 12 l min^−1^. Nozzle voltage was set to 1,500 V, and capillary voltage was set to 3,500 V. Dynamic multiple reaction monitoring was used to quantify analytes, using nitrogen as the collision gas. Lumefantrine was quantified at transitions 528.16 to 510.00 *m*/*z* at 28 collision energy (CE), with a qualifier transition from 528.16 to 383.00 *m*/*z* (40 CE). Halofantrine was used as an internal standard and quantified with the 500.18 to 142.10 *m*/*z* transition (24 CE) and qualified with the 500.18 to 100.10 *m*/*z* transition. All transitions used a cell accelerator voltage of 4. Data analysis was performed with MassHunter B10.1 (Agilent Technologies). Linear calibration curves were weighted by the reciprocal of the standard concentrations used, that is, 1/*x*.

A ten-point calibration curve of halofantrine and lumefantrine was prepared with concentrations ranging from 1 to 2,500 ng ml^−1^. For plasma sample preparation, 250 μl of plasma, 20 μl of halofantrine at 2,500 ng ml^−1^ and 730 μl of 90:10 methanol:tetrahydrofuran was added for protein precipitation. Samples were vortexed and centrifuged at 15,000*g* for 15 min. The resulting supernatant (200 μl) was transferred to glass vials for analysis.

### Lactase activity after dithiol molecule treatment

Lactase (18 µg, 60 µl) was added to DMSA or PEG–dithiol to a final DMSA or PEG–dithiol concentration of 2.5, 5 or 10 mM. Treatments were incubated at 37 °C for 20 min. Lactase activity was assayed by adding 60 μl of 5 mM ONPG and incubation for 1 min at room temperature. Then, 300 μl of 1 M Na_2_CO_3_ was added to stop the reaction, and the absorbance of the solution was read at *λ* = 420 nm.

### Enzyme encapsulation in LIFT hydrogels

The effect of acid on enzyme activity was determined by treating lactase (0.24 mg, 60 µl) with either SGF or PBS and incubating at 37 °C, 50 RPM for various times. Enzymatic activity after incubation was determined by adding 60 μl of 5 mM ONPG and incubation for 1 min at room temperature. After, 300 μl of 1 M Na_2_CO_3_ was added to stop the reaction, and the absorbance of the solution was read at *λ* = 420 nm. For in vitro hydrogel experiments, lactase (0.20 mg) was suspended in 60 μl polymer solution (0.5% alginate/5% w/v four-arm PEG–maleimide) and cast in crosslinker solution (200 mM CaCl_2_/10 mM PEG–dithiol). Alginate hydrogels were prepared in 200 mM CaCl_2_ solution only, and both hydrogels were prepared with and without CaCO_3_ (42.68 mg ml^−1^). Hydrogels were then challenged with 1 ml SGF for 1 h at 37 °C. After acid incubation, enzymatic activity was quantified as above. Similar experiments were performed with α-galactosidase and cellulase, except using X-α-Gal and EnzChek reagents to quantify each respective enzyme activity. For trypsin challenge experiments, lactase-containing hydrogels (60 μl, 0.20 mg lactase) were prepared and incubated with trypsin (40 mg ml^−1^) for 6 h at 37 °C. Free lactase and alginate-only hydrogels were included as controls. Lactase enzyme activity was quantified as previously described, and compared between trypsin-treated samples and naive samples to determine relative absorbance.

Encapsulated lactase activity was tested in rat and porcine models. Rats were fasted overnight before administration. The following day, 3 ml of crosslinker solution (200 mM CaCl_2_/10 mM PEG–dithiol) was administered via oral gavage immediately followed by 1 ml of polymer solution (0.5% alginate/5% w/v four-arm PEG–maleimide with 0.24 mg lactase). Calcium carbonate (42.69 mg) was included either in the crosslinker solution (separate) or in the polymer solution (co-encapsulated). After 1 or 2 h, rats were euthanized, and the hydrogels were collected. Hydrogels were weighed and minced, and enzymatic activity was quantified as described above and normalized by hydrogel mass. Similar experiments with encapsulated cellulase (0.5 mg) were also performed in rat models. Encapsulated lactase activity was also tested in Yorkshire pigs. Hydrogels were administered via endoscopy into the stomach: first, 200 ml of crosslinker (200 mM CaCl_2_/10 mM PEG–dithiol) was administered followed by 20 ml of 0.5% alginate/5% w/v four-arm PEG–maleimide containing lactase (40.45 mg) with or without co-encapsulated CaCO_3_ (2 g). After 6 h, hydrogels were retrieved, and lactase activity was quantified as described above.

### Bacteria encapsulation in LIFT hydrogels

EcN was isolated from commercially available Mutaflor capsules on lysogeny broth (LB)-agar plates (BD 240230). This strain was transformed with the plasmid pAKlux2 (Addgene 14080) to create a constitutively bioluminescent EcN strain. Bioluminescent EcN was routinely cultured on LB-agar plates at 37 °C or in LB in culture tubes shaken at 37 °C containing ampicillin (100 µg ml^−1^). *L. lactis* MG1363 was purchased from Boca Scientific (ELS01363) and was routinely cultured on M17 + 0.5% glucose-agar plates at 30 °C or in M17 + 0.5% glucose in culture tubes at 30 °C without shaking. The bacterial concentration in overnight cultures was determined by measuring the OD_600_, and the cells were pelleted by centrifugation and resuspended in PBS at the target concentration.

To determine bacteria activity in SGF pH 1.77, bacteria (1 × 10^8^ colony-forming units (CFU)) were added to SGF or PBS and incubated for the indicated timepoints. Bioluminescence (EcN) or luminescence (*L. lactis*) from an ATP quantification assay (CellTiter-Glo, Promega) was recorded using a plate reader (Infinite 200, Tecan). For in vitro hydrogel experiments, bacteria (1 × 10^8^ CFU) was suspended in 60 μl polymer solution (0.5% alginate/5% w/v four-arm PEG–maleimide) and cast in 1 ml of crosslinker solution (200 mM CaCl_2_/10 mM PEG–dithiol) with and without CaCO_3_ (7.5 mg ml^−1^). Hydrogels were transferred to 100 µl SGF for 3 h at 37 °C, 100 RPM. After acid incubation, hydrogels were transferred to a white 96-well plate and analysed for luminescence. For EcN survival growth studies, hydrogels were then transferred to 1 ml LB medium and incubated for 4 h, 37 °C, 100 RPM. Then, the supernatant was collected and analysed for bioluminescence.

Encapsulated bacteria activity was tested in porcine models. Hydrogels were administered into the stomach of anaesthetized pigs via endoscopy: first, 200 ml of crosslinker (200 mM CaCl_2_/10 mM PEG–dithiol) was administered followed by 20 ml of 0.5% alginate/5% w/v four-arm PEG–maleimide containing bacteria (1.6 × 10^10^ CFU EcN; 4.1 × 10^9^ CFU *L. lactis*) with or without co-encapsulated CaCO_3_ (2 g). After 6–8 h, hydrogels were retrieved, and bacteria viability was quantified. For EcN, viability (bioluminescence) was recorded using an in vivo imaging system (PerkinElmer). For *L. lactis*, hydrogels were randomly sampled and quantified for ATP using CellTiter-Glo assay (Promega) and a plate reader. These experiments were repeated three times in different pigs.

### Data collection and statistical analysis

Samples and measurements were prepared and collected in a randomized manner. No data points were excluded from analysis. Data collection and analysis were not performed blind to the conditions of the experiments. Tecan i-control plate reader software (version 2.0.10.0) was used for measurement and collection of fluorescence, absorbance and luminescence data. Instron Bluehill software (version 3.11.1209) was used for collection of mechanical testing data.

Agilent MassHunter software (version B10.1) was used to analyse pharmacokinetics data. For all statistical tests, *α* = 0.05 was set for statistical significance. Single comparison tests were performed by a two-tailed Student’s *t*-test, and multiple comparisons were performed using a one-way analysis of variance (ANOVA) with post-hoc Tukey’s multiple comparisons test or two-way ANOVA with post-hoc Šidák’s multiple comparisons test. Statistical analysis was performed using GraphPad Prism (Version 9.3.0). Data distribution was assumed to be normal, but this was not formally tested. No statistical methods were used to pre-determine sample sizes, but our sample sizes are similar to those reported in previous publications^34,35,50^.

### Reporting summary

Further information on research design is available in the Nature Portfolio Reporting Summary linked to this article.

## Online content

Any methods, additional references, Nature Portfolio reporting summaries, source data, extended data, supplementary information, acknowledgements, peer review information; details of author contributions and competing interests; and statements of data and code availability are available at 10.1038/s41563-024-01811-5.

### Supplementary information

Supplementary Information Supplementary Figs. 1–12 and Discussion.

Reporting Summary

Supplementary Table 1 Porcine blood chemistry after LIFT hydrogel administration.

Supplementary Table 2 Cell lines.

Supplementary Table 3 Competing interests for G.T.

Supplementary Table 4 Competing interests for R.L.

### Source data

Source Data Figs. 2–6 Source data for main figures.

## Data Availability

All the data supporting the results in this study are available within the paper and its Supplementary Information. Source data are provided with this paper. Additional data may be requested from the authors.
