# Supplementary material for: Drinkable in situ-forming tough hydrogels for gastrointestinal therapeutics
Source: Nat Mater. 2024 Feb 27;23(9):1292–9. doi: 10.1038/s41563-024-01811-5 (PMC11364503; doi:10.1038/s41563-024-01811-5)
Supplement: Supplementary file 1 — Supplementary Figs. 1–12 and Discussion. [file 41563_2024_1811_MOESM1_ESM.pdf]

nature materials

Article

https://doi.org/10.1038/s41563-024-01811-5

Drinkable in situ-forming tough hydrogels for gastrointestinal therapeutics

In the format provided by the authors and unedited

TABLE OF CONTENTS

Supplementary Figure 1. 1

Supplementary Figure 2. 2

Supplementary Figure 3. 3

Supplementary Figure 4. 4

Supplementary Figure 5. 5

Supplementary Figure 6. 6

Supplementary Figure 7. 7

Supplementary Figure 8. 8

Supplementary Figure 8 Discussion. 8

Supplementary Figure 9. 9

Supplementary Figure 10. 10

Supplementary Figure 11. 11

Supplementary Figure 11 Discussion. 11

Supplementary Figure 12. 12

Supplementary Figure 12 Discussion. 12

Supplementary Information References 14

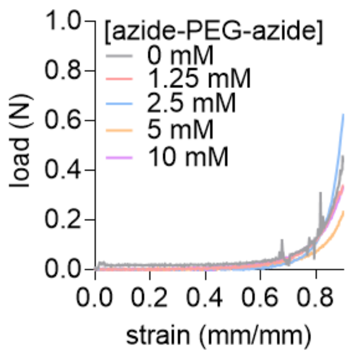

Supplementary Figure 1. Load-strain curves of DBCO-azide PEG/alginate hydrogels. Hydrogels were formed by drop casting a solution of 0.5% alginate/5% w/v 4-arm PEG-DBCO into a solution of 200 mM $CaCl_2$ with the indicated concentrations of PEG-diazide crosslinker. After casting, hydrogels were incubated at 37 °C for 20 min and then mechanically characterized by compression testing. Hydrogels crosslinked in the presence of PEG-diazide were not mechanically different than the alginate control, indicating that the PEG network did not appreciably crosslink within 20 min. Moreover, all hydrogels remained permanently deformed (flattened) after 90% strain.

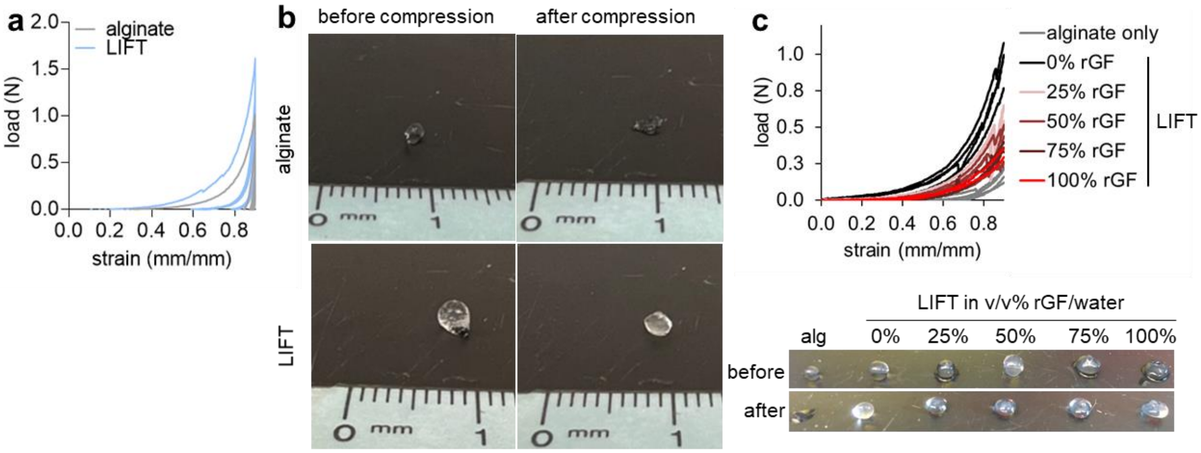

Supplementary Figure 2. Mechanical characterization of LIFT hydrogels crosslinked with DMSA. A. Load-strain curves of LIFT and alginate hydrogels throughout 5 cycles of 90% strain. Hydrogels were formed by drop casting of 0.5% alginate/5% w/v 4-arm PEG-maleimide into a solution of 200 mM $CaCl_2$/10 mM DMSA. After casting, hydrogels were incubated at 37 °C, 50 RPM, 20 min and then mechanically characterized by compression testing. While LIFT hydrogels exhibited some elasticity throughout multiple compressions, alginate hydrogels were permanently deformed after the first compression and were unable to sustain subsequent strains. **B.** Images of alginate and LIFT hydrogels before and after 90% cyclic strain. Whereas alginate hydrogels were permanently deformed, LIFT hydrogels exhibited some recovery. **C.** Top: load-strain curves of LIFT hydrogels after formation in real gastric fluid (rGF). Hydrogels were formed by drop casting of 0.5% alginate/5% w/v 4-arm PEG-maleimide into a solution of rGF with 100 mM $CaCl_2$/5 mM DMSA. To achieve the indicated rGF proportions, rGF was diluted with water. After casting, hydrogels were incubated at 37 °C, 50 RPM, 10 min and then mechanically characterized by compression testing. Despite formation in complex gastric fluid, LIFT hydrogels formed in rGF were mechanically stronger than alginate hydrogels formed in 100 mM $CaCl_2$ without rGF. Bottom: hydrogels before and after 90% strain. While alginate hydrogels remained permanently flattened, LIFT hydrogels formed in rGF retained some degree of their original geometry.

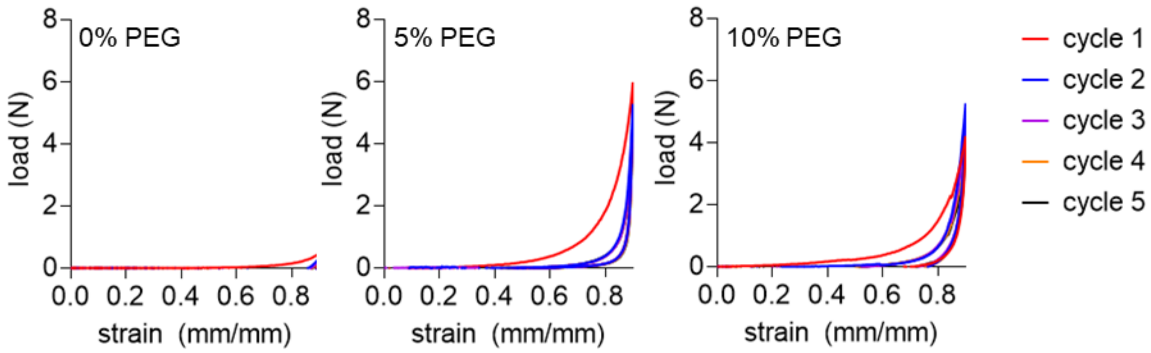

Supplementary Figure 3. Load-strain curves of hydrogels throughout 5 cycles of 90% strain. Hydrogels were formed by drop casting of 0.5% w/v alginate and the indicated amount (% w/v) of 4-arm PEG-maleimide into a solution of 200 mM $CaCl_2$/10 mM PEG-dithiol. After casting, hydrogels were incubated at 37 °C, 50 RPM, 20 min and then mechanically characterized by compression testing. While hydrogels containing a crosslinked PEG network recovered a degree of their original geometry throughout multiple compressions, alginate hydrogels were permanently flattened after the first compression and were unable to sustain subsequent strains.

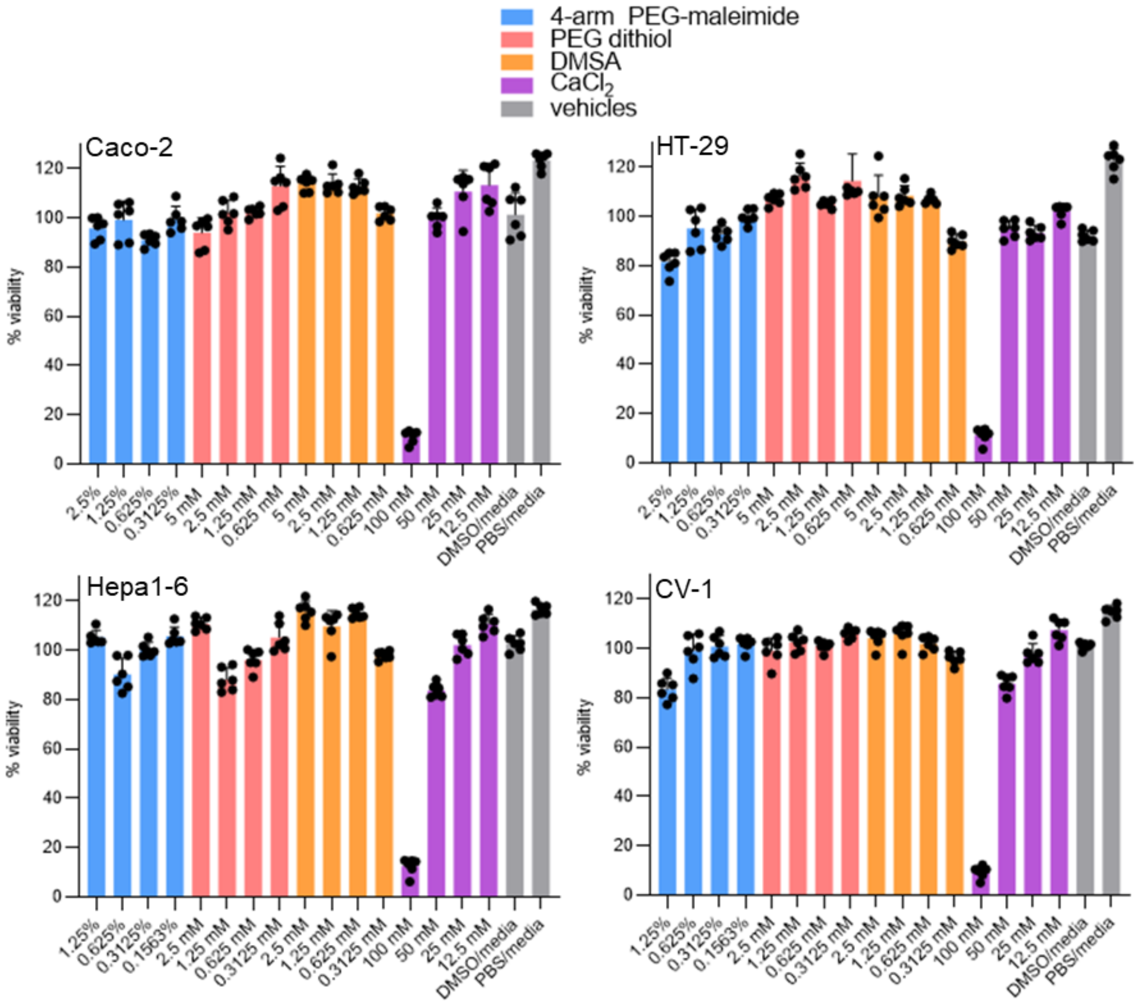

Supplementary Figure 4. Viability of various cell lines after 24 h exposure to various LIFT components. The following reagents were tested: 4-arm PEG-maleimide, PEG dithiol, dimercaptosuccinic acid (DMSA), and $CaCl_2$. Cell lines tested were human colon epithelial Caco-2, human colon epithelial HT-29, mouse liver Hepa1-6, and monkey kidney CV-1 cells. An $n = 6$ wells were tested for each treatment per cell line; viability was normalized to untreated cells. All data are presented as mean ± standard deviation.

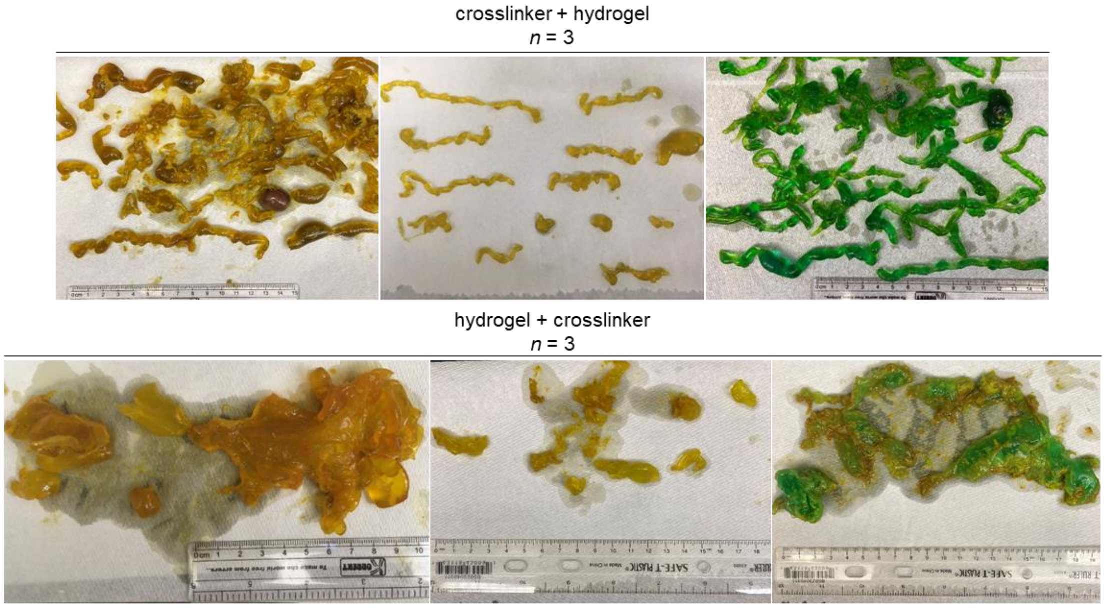

Supplementary Figure 5. Structure study of LIFT hydrogels formed *in vivo*. Top: Female Yorkshire pigs (*n* = 3) were administered crosslinker solution (200 mM $CaCl_2$/10 mM DMSA) followed by hydrogel solution (0.5% alginate/5% w/v 4-arm PEG-maleimide). In some experiments, green dye was added for color contrast. Bottom: female Yorkshire pigs (*n* = 3) were administered hydrogel solution followed by crosslinker solution. In some experiments, green dye was added for color contrast. Generally, the administration order crosslinker + hydrogel resulted in consistent "noodle-like" hydrogels, while hydrogel + crosslinker resulted in more heterogenous hydrogel shapes.

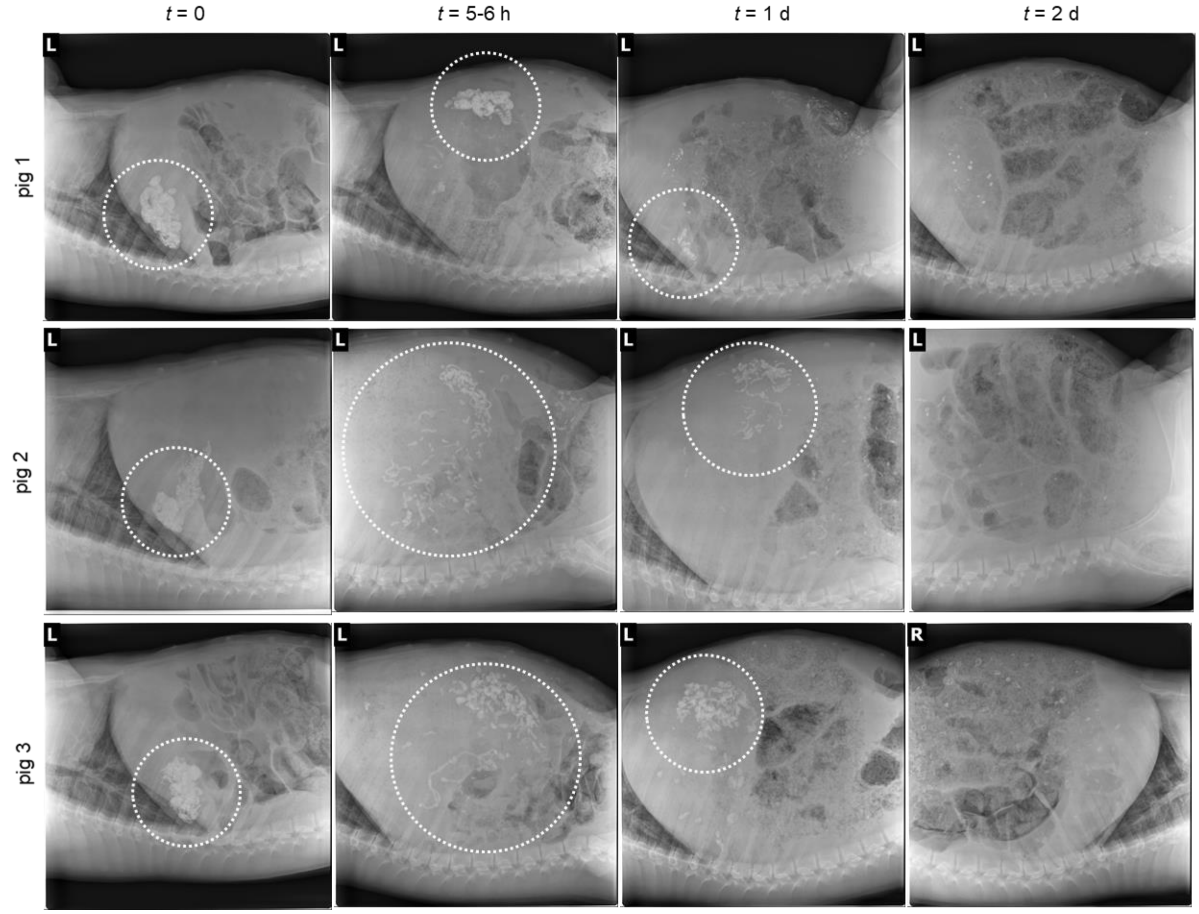

Supplementary Figure 6. *In vivo* retention of LIFT hydrogels. Female Yorkshire pigs (n = 3) were administered a solution of crosslinker (200 mM $CaCl_2$/10 mM PEG-dithiol) followed by polymer (0.5% alginate/5% w/v 4-arm PEG-maleimide) loaded with 20% w/v $BaSO_4$ to facilitate X-ray imaging. Hydrogels were present within the stomach up to 24 h after administration.

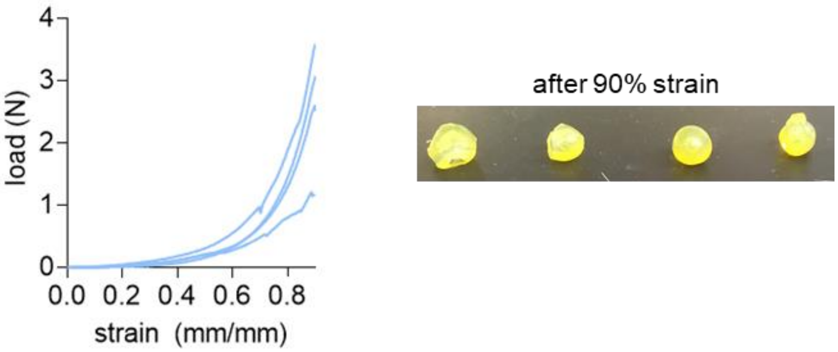

Supplementary Figure 7. Mechanical characterization of LIFT hydrogels, crosslinked with DMSA, formed *in vivo*. Left: load-strain curves of LIFT hydrogels after 90% strain. Hydrogels were formed *in vivo* in female Yorkshire pigs by administration of a crosslinker solution (200 mM $CaCl_2$/10 mM DMSA) followed by polymer solution (0.5% alginate/5% w/v 4-arm PEG-maleimide). Hydrogels were retrieved 6-8 h after administration. Right: images of LIFT hydrogels after 90% strain.

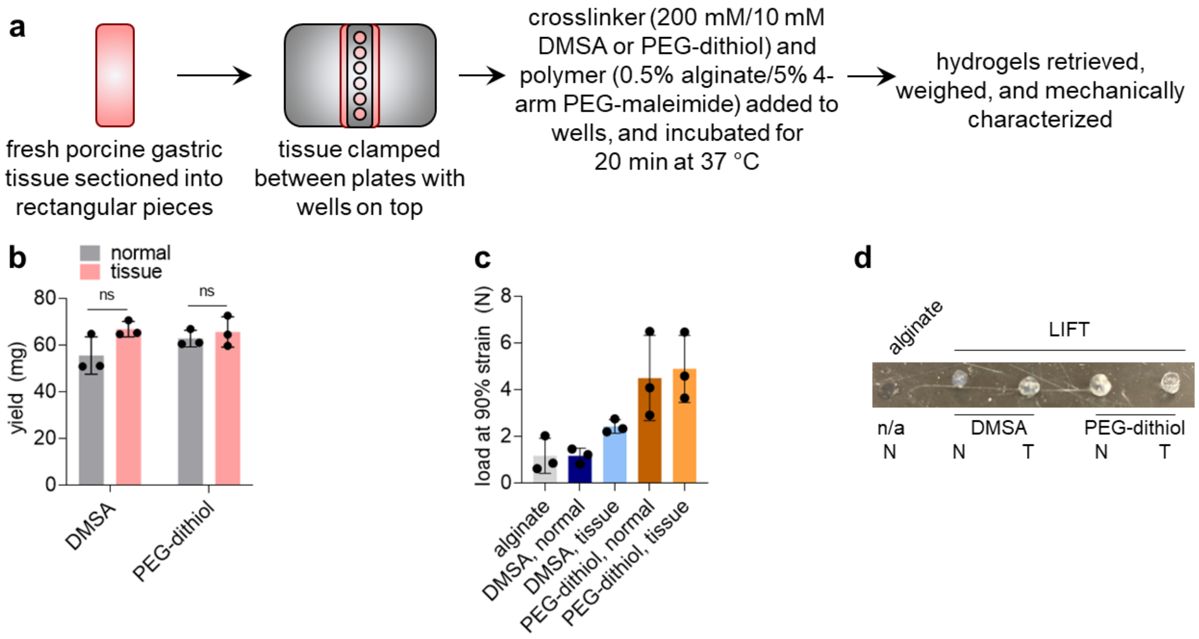

Supplementary Figure 8. LIFT hydrogel formation and performance in the presence of gastric tissue. A. Experimental setup. Fresh gastric tissue was sectioned and clamped between plates to facilitate addition and formation of LIFT hydrogels, which were characterized for yield (mass) and mechanical behavior. **B.** Yield, in mass, of LIFT hydrogels formed in normal plastic plate wells or gastric tissue environment. Both dithiol crosslinkers DMSA and PEG-dithiol were examined. Statistical analysis was performed by two-way ANOVA with post-hoc Sidak's multiple comparisons test, n = 3 independent experiments. **C.** Loads experienced by alginate or LIFT hydrogels at 90% strain, n = 3 independent experiments. **D.** Images of hydrogels formed in normal plastic plate wells (N) or gastric tissue environment (T) after 90% strain. All data are presented as mean ± standard deviation.

Supplementary Figure 8 Discussion. Gastrointestinal mucus is abundant with cysteines, which may react with thiol and maleimide groups present within LIFT. This may impact hydrogel yield or cause hydrogel adhesion to gastric tissue, as has been leveraged in other systems[1]. To test the impact of tissue on hydrogel yield, crosslinker was applied directly to tissue or a plastic plate as a control. We utilized a modified Franz diffusion device, similar to what we have previously reported, to create individual "wells" on top of stomach tissue for this experiment[2] (**Supplementary Figure 8a**). Hydrogel yield, defined by mass, did not significantly differ between formation in a gastric tissue environment or normal plastic plate, regardless of whether DMSA or PEG-dithiol was used (**Supplementary Figure 8b**). Moreover, formation in a gastric tissue environment did not appear to negatively impact hydrogel mechanical properties (**Supplementary Figure 8c, Supplementary Figure 8d**). Thus, side reactions with tissue do not seem to occur at a scale that significantly impacts hydrogel formation. LIFT adhesion to tissue was tested using a tilt test. After incubation, tilting, and washing of wells, we did not observe LIFT adhesion to gastric tissue. This observation aligns with our *in vivo* porcine experiments, in which we do not observe hydrogel adhesion after endoscopic delivery, and may be due to saturation of tissue thiols and/or PEG-maleimide groups with crosslinker thiols.

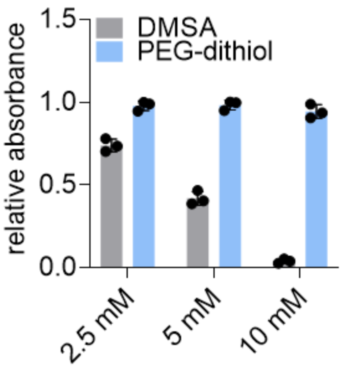

Supplementary Figure 9. Lactase activity after exposure to dithiol compounds. Lactase was added to either DMSA or PEG-dithiol at the indicated concentrations and incubated at 37 °C, 50 RPM, 20 min. Lactase activity was quantified by addition of ONPG and analysis of the colored product. Absorbance was normalized to an untreated lactase control, *n* = 3 independent experiments. Data is presented as mean ± standard deviation.

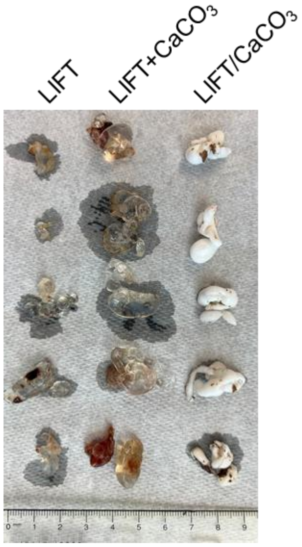

Supplementary Figure 10. LIFT hydrogels after formation in rats. Male Sprague-Dawley rats were orally gavaged with a crosslinker solution (200 mM $CaCl_2$/10 mM PEG-dithiol) followed by a polymer solution (0.5% alginate/5% w/v 4-arm PEG-maleimide) with or without $CaCO_3$.

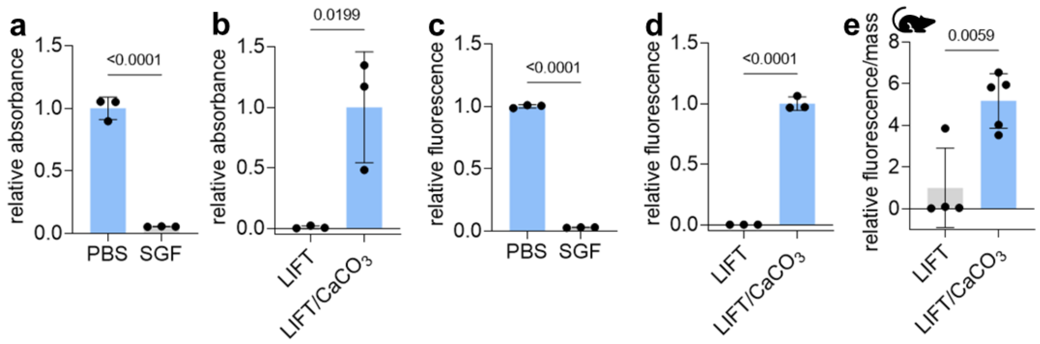

Supplementary Figure 11. LIFT hydrogel co-encapsulation of $CaCO_3$ protects enzyme activity in acid. A. α-galactosidase activity, as measured by X-α-gal assay, after 10 min incubation in PBS or SGF at 37 °C. Absorbances were normalized to that of α-galactosidase incubated in PBS, $n = 3$ independent experiments. **B.** α-galactosidase activity after hydrogel encapsulation with or without $CaCO_3$ co-encapsulation and incubation in SGF for 1 h, . Absorbances were normalized to that of LIFT/$CaCO_3$, $n = 3$ independent experiments. **C.** Cellulase activity, as measured by EnzChek substrate, after 10 min incubation in PBS or SGF at 37 °C. Fluorescence was normalized to that of cellulase incubated in PBS, $n = 3$ independent experiments. **D.** Cellulase activity after hydrogel encapsulation with or without $CaCO_3$ co-encapsulation and incubation in SGF for 1 h. Fluorescence was normalized to that of LIFT/$CaCO_3$, $n = 3$ independent experiments. **E.** Activity of cellulase encapsulated in LIFT hydrogels after 1 h in female Sprague-Dawley rats with or without $CaCO_3$ co-encapsulation. Fluorescence was normalized by hydrogel mass; $n = 4$ (LIFT) or 5 (LIFT/$CaCO_3$) rats were tested. For these set of experiments, statistical tests were performed with a two-tailed Student's *t*-test. All data are presented as mean ± standard deviation.

Supplementary Figure 11 Discussion. To test if LIFT is broadly compatible with enzyme encapsulation, we conducted additional experiments with α-galactosidase and cellulase, two important enzyme supplements for patients with digestive enzyme deficiencies or irritable bowel syndrome[3, 4]. Short incubations in gastric acid significantly degraded enzyme activity (**Supplementary Figure 11a, Supplementary Figure 11c**). In vitro, LIFT/CaCO3 protected both enzymes against acid-triggered inactivation (**Supplementary Figure 11b, Supplementary Figure 11d**); further experiments in rats also demonstrated that LIFT/CaCO3 could protect cellulase activity after 1 h in rat stomachs (**Supplementary Figure 11e**).

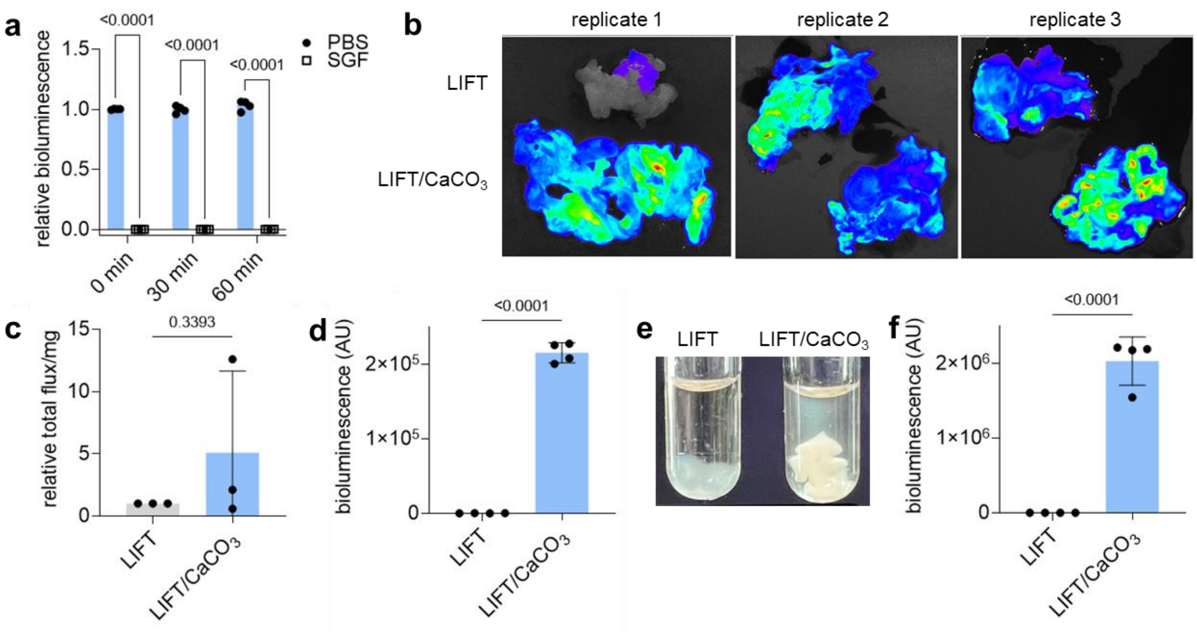

Supplementary Figure 12. LIFT hydrogel co-encapsulation of $CaCO_3$ protects *E. coli* Nissle 1917 activity in simulated gastric fluid. A. Bioluminescence of luciferase-expressing *E. coli* Nissle 1917 bacteria after various incubations times in PBS or SGF at 37 °C. Bioluminescence was normalized to that of bacteria incubated in PBS at each time point. Statistical analysis was performed by two-way ANOVA with post-hoc Sidak's multiple comparisons test, $n = 3$ independent experiments. **B.** Bioluminescence of bacteria encapsulated in LIFT hydrogels with and without $CaCO_3$. Female Yorkshire pigs ($n = 3$) were administered a solution of crosslinker (200 mM $CaCl_2$/10 mM PEG-dithiol) followed by polymer (0.5% alginate/5% w/v 4-arm PEG-maleimide) loaded with 1.6×10^{10} CFU luciferase-expressing bacteria. **C.** After 6-8 h, hydrogels were retrieved and bioluminescence was measured, normalized to hydrogel mass and to control hydrogels without $CaCO_3$. Statistical analysis was performed by two-tailed Student's *t*-test, $n = 3$ independent pig experiments. **D.** Bioluminescence of bacteria encapsulated in LIFT hydrogels with or without $CaCO_3$ and incubated in SGF for 3 h. Statistical analysis was performed by two-tailed Student's *t*-test, $n = 4$ independent experiments. **E.** Representative images of culture tubes containing LB culture medium after bacteria-containing LIFT hydrogels were challenged with SGF and then incubated in the culture tubes for 4 h at 37 °C. Cloudiness of medium indicates viable bacteria remaining inside the hydrogel. **F.** Bioluminescence quantification of media supernatants from panel E. Statistical analysis was performed by two-tailed Student's *t*-test, $n = 4$ independent experiments. All data are presented as mean ± standard deviation.

Supplementary Figure 12 Discussion. Here, we utilized an engineered *E. coli* Nissle 1917 (EcN) that expresses both luciferase and luciferin; bioluminescence has been utilized to rapidly query bacterial viability and metabolism[5]. This particular strain was selected due to its safety and wide use as a chassis for synthetic biology therapeutics[6]. Indeed, even short incubations of EcN in SGF pH 1.77 resulted in significant decreases in bioluminescence (**Supplementary Figure 12a**). We tested the capacity of LIFT hydrogels to protect bacterial activity in porcine models after 6-8 h incubation in the stomach. While bacteria encapsulated in LIFT/$CaCO_3$ hydrogels exhibited greater average bioluminescence than those in LIFT hydrogels, this difference did not reach

statistical significance (**Supplementary Figure 12b, Supplementary Figure 12c**). The relatively high pH values of pig gastric fluid (pH > 5) may not be sufficient to reduce EcN viability[7]. We reasoned that in terms of acidity, *in vitro* studies may provide a more aggressive challenge than *in vivo* treatment in pigs that is physiologically relevant to human gastric fluid (pH 1.4-2.1 in the fasted state)[8]. Therefore, we asked if LIFT hydrogels were capable of protecting bacterial bioluminescence upon encapsulation with or without $CaCO_3$ and after challenge with SGF pH 1.77 for 3 h. Only co-encapsulation with $CaCO_3$ resulted in bacterial bioluminescence compared to LIFT hydrogel control (**Supplementary Figure 12d**). To further confirm that bacteria were indeed viable and capable of growth, hydrogels were incubated in growth media after SGF challenge. While the media of LIFT hydrogels remained clear, the media of LIFT/$CaCO_3$ hydrogels appeared turbid, suggesting bacterial proliferation (**Supplementary Figure 12e**). The supernatant was quantified for bioluminescence to confirm bacterial viability and metabolism; only supernatant from LIFT/$CaCO_3$ hydrogels exhibited bioluminescence (**Supplementary Figure 12f**).

Supplementary Information References

1. Tonglairoum P, Brannigan RP, Opanasopit P, Khutoryanskiy VV. Maleimide-bearing nanogels as novel mucoadhesive materials for drug delivery. *J Mater Chem B* 2016, **4**(40)**:** 6581-6587.

2. von Erlach T, Saxton S, Shi Y, Minahan D, Reker D, Javid F, *et al.* Robotically handled whole-tissue culture system for the screening of oral drug formulations. *Nat Biomed Eng* 2020, **4**(5)**:** 544-559.

3. Tuck CJ, Taylor KM, Gibson PR, Barrett JS, Muir JG. Increasing Symptoms in Irritable Bowel Symptoms With Ingestion of Galacto-Oligosaccharides Are Mitigated by alpha-Galactosidase Treatment. *Am J Gastroenterol* 2018, **113**(1)**:** 124-134.

4. Ran ZH, Yuan YZ, Li ZS, Wang JY, Zong CH, Xie WF, *et al.* The efficacy of Combizym in the treatment of Chinese patients with dyspepsia: a multicenter, randomized, placebo-controlled and cross-over study: Shanghai Combizym Clinical Cooperative Group. *J Dig Dis* 2009, **10**(1)**:** 41-48.

5. Anselmo AC, McHugh KJ, Webster J, Langer R, Jaklenec A. Layer-by-Layer Encapsulation of Probiotics for Delivery to the Microbiome. *Adv Mater* 2016, **28**(43)**:** 9486-9490.

6. Kurtz CB, Millet YA, Puurunen MK, Perreault M, Charbonneau MR, Isabella VM, *et al.* An engineered E. coli Nissle improves hyperammonemia and survival in mice and shows dose-dependent exposure in healthy humans. *Sci Transl Med* 2019, **11**(475).

7. Merchant HA, McConnell EL, Liu F, Ramaswamy C, Kulkarni RP, Basit AW, *et al.* Assessment of gastrointestinal pH, fluid and lymphoid tissue in the guinea pig, rabbit and pig, and implications for their use in drug development. *Eur J Pharm Sci* 2011, **42**(1-2)**:** 3-10.

8. Yang SY, Sencadas V, You SS, Jia NZ, Srinivasan SS, Huang HW, *et al.* Powering Implantable and Ingestible Electronics. *Adv Funct Mater* 2021, **31**(44).
