# Supplementary material for: Drinkable in situ-forming tough hydrogels for gastrointestinal therapeutics
Source: Nat Mater. 2024 Feb 27;23(9):1292–9. doi: 10.1038/s41563-024-01811-5 (PMC11364503; doi:10.1038/s41563-024-01811-5)
Supplement: Supplementary file 2 — Reporting Summary [file 41563_2024_1811_MOESM2_ESM.pdf]

nature portfolio

Corresponding author(s): Giovanni Traverso

Last updated by author(s): Oct 19, 2023

Reporting Summary

Nature Portfolio wishes to improve the reproducibility of the work that we publish. This form provides structure for consistency and transparency in reporting. For further information on Nature Portfolio policies, see our Editorial Policies and the Editorial Policy Checklist.

Statistics

For all statistical analyses, confirm that the following items are present in the figure legend, table legend, main text, or Methods section.

n/a	Confirmed	
☐	☒	The exact sample size (n) for each experimental group/condition, given as a discrete number and unit of measurement
☐	☒	A statement on whether measurements were taken from distinct samples or whether the same sample was measured repeatedly
☐	☒	The statistical test(s) used AND whether they are one- or two-sided<br>*Only common tests should be described solely by name; describe more complex techniques in the Methods section.*
☒	☐	A description of all covariates tested
☐	☒	A description of any assumptions or corrections, such as tests of normality and adjustment for multiple comparisons
☐	☒	A full description of the statistical parameters including central tendency (e.g. means) or other basic estimates (e.g. regression coefficient) AND variation (e.g. standard deviation) or associated estimates of uncertainty (e.g. confidence intervals)
☐	☒	For null hypothesis testing, the test statistic (e.g. F, t, r) with confidence intervals, effect sizes, degrees of freedom and P value noted<br>*Give P values as exact values whenever suitable.*
☒	☐	For Bayesian analysis, information on the choice of priors and Markov chain Monte Carlo settings
☒	☐	For hierarchical and complex designs, identification of the appropriate level for tests and full reporting of outcomes
☒	☐	Estimates of effect sizes (e.g. Cohen's d, Pearson's r), indicating how they were calculated

Our web collection on statistics for biologists contains articles on many of the points above.

Software and code

Policy information about availability of computer code

Data collection	Tecan i-control plate reader software (version 2.0.10.0) was used for measurement and collection of fluorescence, absorbance and luminescence data. Instron Bluehill software (version 3.11.1209) was used for collection of mechanical testing data.
Data analysis	GraphPad Prism (Version 9.3.0) was used for statistical analysis of data. Agilent MassHunter software (version B10.1) was used to analyze pharmacokinetics data.

For manuscripts utilizing custom algorithms or software that are central to the research but not yet described in published literature, software must be made available to editors and reviewers. We strongly encourage code deposition in a community repository (e.g. GitHub). See the Nature Portfolio guidelines for submitting code & software for further information.

Data

Policy information about availability of data

All manuscripts must include a data availability statement. This statement should provide the following information, where applicable:

- Accession codes, unique identifiers, or web links for publicly available datasets
- A description of any restrictions on data availability
- For clinical datasets or third party data, please ensure that the statement adheres to our policy

All the data supporting the results in this study are available within the paper and its Supplementary Information. Source data for main figures are provided within the paper. Additional data may be requested from the authors.

Human research participants

Policy information about studies involving human research participants and Sex and Gender in Research.

Reporting on sex and gender	N/A
Population characteristics	N/A
Recruitment	N/A
Ethics oversight	N/A

Note that full information on the approval of the study protocol must also be provided in the manuscript.

Field-specific reporting

Please select the one below that is the best fit for your research. If you are not sure, read the appropriate sections before making your selection.

☒ Life sciences ☐ Behavioural & social sciences ☐ Ecological, evolutionary & environmental sciences

For a reference copy of the document with all sections, see nature.com/documents/nr-reporting-summary-flat.pdf

Life sciences study design

All studies must disclose on these points even when the disclosure is negative.

Sample size	Sample sizes for animal studies were based on prior work (Refs. J. Liu, et al. Nat. Commun., 2017; A. C. Anselmo, et al. Adv. Mater. 2016; X. Liu, et al. Nat. Commun. 2019) without using statistical methods to pre-determine sample sizes.
Data exclusions	No data were excluded.
Replication	All experimental data were replicated at least 3 times in independent experiments.
Randomization	Animals were randomized to treatment groups. Samples and measurements were prepared and collected in a randomized manner.
Blinding	Data collection and analysis were not performed blind to the conditions of the experiments, because the same scientists designed and carried out the experiments.

Reporting for specific materials, systems and methods

We require information from authors about some types of materials, experimental systems and methods used in many studies. Here, indicate whether each material, system or method listed is relevant to your study. If you are not sure if a list item applies to your research, read the appropriate section before selecting a response.

Materials & experimental systems

n/a	Involved in the study	
☒	☐	Antibodies
☐	☒	Eukaryotic cell lines
☒	☐	Palaeontology and archaeology
☐	☒	Animals and other organisms
☒	☐	Clinical data
☒	☐	Dual use research of concern

Methods

n/a	Involved in the study	
☒	☐	ChIP-seq
☒	☐	Flow cytometry
☒	☐	MRI-based neuroimaging

Eukaryotic cell lines

Policy information about cell lines and Sex and Gender in Research

Cell line source(s)	All cell lines were sourced from ATCC. From ATCC: HT-29 was sourced from a female patient; Caco-2 was sourced from a male patient; Hepa 1-6 was sourced from a C57/L mouse, and CV-1 was sourced from a male adult African green monkey.

Authentication	HT-29 and Caco-2 cells were authenticated by ATCC using Short Tandem Repeat (STR) analysis as described in 2012 in ANSI Standard ASN-0002. Hepa1-6 and CV-1 cells were not authenticated.
Mycoplasma contamination	All cell lines tested negative for mycoplasma.
Commonly misidentified lines (See ICLAC register)	N/A

Animals and other research organisms

Policy information about studies involving animals; ARRIVE guidelines recommended for reporting animal research, and Sex and Gender in Research

Laboratory animals	Female Yorkshire pigs aged 3-7 months (50-100 kg, sourced from Animal Biotech Industries, Inc. or Tufts University School of Veterinary Medicine) and male or female Sprague-Dawley rats (>400 g, sourced from Charles River, strain code 001) were used. Due to limited supplies of large >400 g rats, rats were used regardless of age.
Wild animals	The study did not involve wild animals.
Reporting on sex	Data reported in this study are not sex-specific and therefore no sex-specific analysis was conducted.
Field-collected samples	The study did not involve samples collected from the field.
Ethics oversight	All animal studies were performed only after Massachusetts Institute of Technology Committee on Animal Care review and ethical approval and under veterinary supervision. The Massachusetts Institute of Technology Division of Comparative Medicine provided guidance and training.

Note that full information on the approval of the study protocol must also be provided in the manuscript.
