# Supplementary material for: Drinkable in situ-forming tough hydrogels for gastrointestinal therapeutics
Source: Nat Mater. 2024 Feb 27;23(9):1292–9. doi: 10.1038/s41563-024-01811-5 (PMC11364503; doi:10.1038/s41563-024-01811-5)
Supplement: Supplementary file 3 — Porcine blood chemistry after LIFT hydrogel administration. [file 41563_2024_1811_MOESM3_ESM.pdf]

Supplementary Table 1. Porcine blood chemistry after LIFT hydrogel administration

time	ALP (U/L)	AST (U/L)	ALT (U/L)	BUN (mg/dL)	creatinine (mg/dL)
baseline	131.0 ± 25.7	18.7 ± 4.0	41.7 ± 6.8	8.7 ± 0.6	1.1 ± 0.1
24 h	132.3 ± 22.5	22.3 ± 17.2	41.7 ± 6.1	12.7 ± 4.5	1.2 ± 0.2
48 h	115.0 ± 16.5	22.3 ± 5.5	38.7 ± 5.9	9.0 ± 1.7	1.1 ± 0.2

ALP, alkaline phosphatase; AST, aspartate aminotransferase; ALT, alanine transaminase; BUN, blood urea nitrogen. Baseline: sample was collected before LIFT hydrogel administration. An n = 3 pigs were tested.
