# Supplementary material for: Drinkable in situ-forming tough hydrogels for gastrointestinal therapeutics
Source: Nat Mater. 2024 Feb 27;23(9):1292–9. doi: 10.1038/s41563-024-01811-5 (PMC11364503; doi:10.1038/s41563-024-01811-5)
Supplement: Supplementary file 4 — Cell lines. [file 41563_2024_1811_MOESM4_ESM.pdf]

Supplementary Table 2. Cell lines.

cell line	vendor	ICLAC misidentified cell line?	authenticated?
Caco-2	ATCC HTB-37	no	authenticated, 2021-04-20
HT-29	ATCC HTB-38	no	authenticated, 2022-04-15
Hepa1-6	ATCC CRL-1830	no	did not submit for authentication
CV-1	ATCC CCL-70	no	ATCC does not provide authentication services for non-human nor non-mouse cells

ATCC, American Type Culture Collection; ICLAC, International Cell Line Authentication Committee, checked using Version 12 Table 1 (release date 2023-01-16); authentication was performed as follows by ATCC:

Seventeen short tandem repeat (STR) loci plus the gender determining locus, Amelogenin, were amplified using the commercially available PowerPlex® 18D Kit from Promega. The cell line sample was processed using the ABI Prism® 3500xl Genetic Analyzer. Data were analyzed using GeneMapper® ID-X v1.2 software (Applied Biosystems). Appropriate positive and negative controls were run and confirmed for each sample submitted.
