# Supplementary material for: Drinkable in situ-forming tough hydrogels for gastrointestinal therapeutics
Source: Nat Mater. 2024 Feb 27;23(9):1292–9. doi: 10.1038/s41563-024-01811-5 (PMC11364503; doi:10.1038/s41563-024-01811-5)
Supplement: Supplementary file 5 — Competing interests for G.T. [file 41563_2024_1811_MOESM5_ESM.pdf]

Disclosures - Carlo Giovanni Traverso - encompassing all professional not-for-profit and for profit relationships
Date: March 7, 2023

Please note that I make every effort to maintain this document up to date and as part of the disclosure process governed by the Sunshine Act further details can be found at:
https://openpaymentsdata.cms.gov/physician/431893

Employment and Professional Affiliations	**Position(s)**	**Years**
Trinity College, University of Cambridge	Undergraduate student (1995-1998), Junior Research Fellow / Title A (2002-2007), Medical Student (2002-2006)	1995-1998, 2002-2007
Hospital for Sick Children, University of Toronto	Research Assistant (1997-1999)	1997-1999
Johns Hopkins University	PhD graduate student (1998-2002), Post-doctoral fellow (2002-2003)	1998-2003
Harvard Medical School	Fellow (2007-2014), Instructor (2014-2017), Assistant Professor, part time (2017-present)	2007-present
Brigham and Women's Hospital	Internal Medicine Resident (2007-2009), Attending Physician (2016-present)	2007-2009, 2016-present
Massachusetts Institute of Technology	Research Affiliate (2009-2018), Visiting Scientist (2018-present), Assistant Professor (2019-present)	2009-present
Massachusett General Hospital	Gastroenterology Fellow (2009-2014), Attending Physician (2014-2015)	2009-2015

For Profit	**Equity/Stock**	**Board/Advisor**	**Consultant**	**Royalties**	**Gifts**	**Grants/ Scholarship**	**Years**
Exact Sciences				x			2002-
Horizon				x			2014-
Pavoda	x	x	x				2014-2016
Entrega, Inc			x				2015
CBSET			x				2015
Avaxia			x				2015
Lyndra	x	x		x			2015-present
Novo Nordisk			x			x	2015-present
SNS Nano			x				2015-2016
Hoffman la Roche						x	2015-2017
Janssen							2016
Egalet		x	x				2016
Janssen			x				2016
BMS					x		2016
Synlogic			x				2016-2018
Freenome						x	2016-2018
Suono Bio	x	x		x			2017-present
Merck			x				2018
Verily			x				2018-2019
Eagle Pharmaceuticals, Inc			x				2018-present
Vivtex	x	x		x			2018-present
Celero Systems	x	x		x			2018-present
Bilayer Therapeutics, Inc	x	x		x			2020-present
Teal Bio, Inc	x	x		x			2020-present
Oracle						x	2020-2021
Wired Consulting			x				2020
Avadel Pharmaceuticals			x				2021
Moderna			x				2022-present
Syntis Bio	x	x	x				2022-present
Vitakey	x	x	x				2023-present
CSL Vifor						x	2022-present

Not for Profit	**Equity/Stock**	**Board/Advisor**	**Consultant**	**Royalties**	**Gifts**	**Grants/ Scholarship**	Years
Draper Laboratory						x	2015-2017
MIT Lincoln Laboratory						x	2012, 2019-2020
NIH/NIBIB						x	2018-present
Kenneth Rainin		x					2015-2016
Bill and Melinda Gates Foundation					`	x	2013-present
NIH/NCI						x	2018-present
Johns Hopkins University / Technology Transfer Office				x			2002-present
Massachusetts Institute of Technology / Technology Licensing Office				x			2015-present
Mass General Brigham Innovation / Technology Licensing				x			2018-present
Cambridge Commonwealth and Overseas Trusts, Univ. of Cambridge						x	1995-1998
Foulkes Foundation						x	2003-2006
Trinity College, University of Cambridge						x	2003-2006
The Leona M. and Harry B. Helmsley Charitable Trust						x	2019-2022
Karl van Tassel (1925) Career Development Professorship, MIT						x	2020-present
Defense Advanced Research Projects Agency						x	2021-present
