# Supplementary material for: Drinkable in situ-forming tough hydrogels for gastrointestinal therapeutics
Source: Nat Mater. 2024 Feb 27;23(9):1292–9. doi: 10.1038/s41563-024-01811-5 (PMC11364503; doi:10.1038/s41563-024-01811-5)
Supplement: Supplementary file 6 — Competing interests for R.L. [file 41563_2024_1811_MOESM6_ESM.pdf]

2023 Competing Interests Disclosure

From FY 2018 to the present, Dr. Robert Langer receives licensing fees (to patents in which he was an inventor on) from, invested in, consults (or was on Scientific Advisory Boards or Boards of Directors) for, lectured (and received a fee), or conducts sponsored research at MIT for which he was not paid for the following entities:

1. 611 Therapeutics
2. Abpro International;
3. Acorda (Formerly Civitas Therapeutics);
4. Alfred University;
5. Aleph Farms;
6. Alivio Therapeutics;
7. Alkermes;
8. Allevi;
9. Allurion;
10. Alnylam Pharmaceuticals, Inc;
11. Amberstone Bioscience;
12. Amgen;
13. aMoon
14. Apotex;
15. Arcadia Biosciences, Inc;
16. Arsenal Medical;
17. Artificial Cell Technology, Inc;
18. Avalon-Globocare;
19. Bai Biosciences;
20. BASF Corporation;
21. Bayer;
22. Balzan Foundation;
23. Bexson Biomedical
24. Bilayer Therapeutics;
25. Biogen;
26. BioInnovation Institute (Novo Nordisk Founden);
27. BioTE Medical;
28. Blackrock;
29. Blackstone (Formerly Clarus);
30. Boston Children's Hospital;
31. CBC Group Investment Mgmt Group;
32. Celanese;
33. Celero;
34. Cellink/BICO
35. Cellomics Technology, Llc;
36. Cellular Biomedical;
37. CE&N/ ACS
38. Charles River Laboratories, Inc.;
39. Clontech Laboratories;
40. Combined Therapeutics ("CTx");
41. Conference Forum;
42. Cornell University;
43. Crispr Therapeutics Ag;
44. Crown Bioscience Inc.;
45. Daré Biosciences (Formerly Microchips Biotech, Juniper Pharmaceuticals, and Columbia Laboratories);
46. Daros, Inc.
47. DeepBiome;
48. Dewpoint Therapeutics;
49. Dispendix;
50. Eagle Pharmaceuticals;
51. Earli;
52. Edigene Biotechnology, Inc.;
53. Editas Medicine, Inc.;
54. ELC (Estee Lauder Companies);
55. Eli Lilly;
56. Eisai Inc.;
57. Entrega;
58. EpiBone
59. Establishment Labs, SA.
60. Everlywell;
61. Evox Therapeutics, Ltd.;
62. Fate
63. Flagship Pioneering;
64. Frequency Therapeutics, Inc.;
65. GeneLeap Biotech
66. Genemedicine Co Lmtd;
67. GenScript USA Inc;

2023 Competing Interests Disclosure

From FY 2018 to the present, Dr. Robert Langer receives licensing fees (to patents in which he was an inventor on) from, invested in, consults (or was on Scientific Advisory Boards or Boards of Directors) for, lectured (and received a fee), or conducts sponsored research at MIT for which he was not paid for the following entities:

68. Geneo Medicine;
69. GENUV;
70. Glaxosmithkline Llc;
71. Glycobia;
72. Glympse Bio;
73. Goldman Sachs
74. Greenlight Biosciences;
75. HCR (HealthCare Royalty Partners);
76. HKF DNA Technologies;
77. Hopewell Therapeutics;
78. Horizon Discovery Group Plc;
79. Humacyte, Inc.;
80. IBEX Pharmaceuticals, Inc.;
81. Immunai;
82. ImmuneXcite Inc.;
83. Institute of Immunology Co. Ltd;
84. Integrated DNA Technologies, Inc.;
85. InVivo Therapeutics;
86. IxBio;
87. J.R. Simplot Company;
88. Jnana Therapeutics;
89. Kala Pharmaceuticals;
90. Kallyope, Inc.;
91. Kendall Capital;
92. Kensa;
93. Kodikaz Therapeutics;
94. KAST (Korean Academy of Science and Technology);
95. Ksq Therapeutics, Inc.;
96. Kunlun Capital;
97. Landsdowne Labs;
98. LikeMinds;
99. Lonza;
100. Luminopia, Inc.;
101. Luye (Shandong luye);
102. Lyndra Therapeutics;
103. Lyra Therapeutics (Formerly "480 Biomedical");
104. Maurice Marie Janot Award 2020
105. McGovern Institute;
106. Medikinetics Co., Ltd.;
107. Merck;
108. MGH Ragon Institute;
109. Micelle;
110. Moderna Therapeutics;
111. Momenta;
112. Muse Biotechnologies Inc.
113. Mylan;
114. N2Tech;
115. Nanobiosym;
116. Nanobiotix;
117. Neochromosone;
118. Neoteny 4 LLP;
119. NextRNA;
120. Newbridge Ventures LLC;
121. Noveome Biotherapeutics, Inc.;
122. Novo Nordisk;
123. Ohio State University;
124. Olivo (acquired by Shiseido)
125. Ovid Therapeutics;
126. Particles for Humanity;
127. Pfizer, Inc.;
128. Pioneer Hi-Bred International, Inc.;
129. Placon Therapeutics
130. Polaris Partners;
131. Pontifical Academy of Sciences;
132. Portal Instruments;
133. Preceres, Llc (Acquired by Monsanto);
134. PrognomIQ Inc.;
135. Pulmatrix;

2023 Competing Interests Disclosure

From FY 2018 to the present, Dr. Robert Langer receives licensing fees (to patents in which he was an inventor on) from, invested in, consults (or was on Scientific Advisory Boards or Boards of Directors) for, lectured (and received a fee), or conducts sponsored research at MIT for which he was not paid for the following entities:

136. PureTech;
137. Quris
138. ReLive;
139. Rensselaer Polytechnic Institute/ Department of Chemical and Biological Engineering
140. Reprocell Usa, Inc. (Formerly Stemgent);
141. Replay Bio;
142. Rubius Therapeutics;
143. Satellite Bio;
144. SBEF (Seoul Bio Economy Forum);
145. Secant Medical, Inc.;
146. Seer, Inc.;
147. Selecta Biosciences;
148. Senses LLC;
149. Setsuro Tech Inc.;
150. Seventh Sense Biosystems, Inc.;
151. Shenzhen Rice Life Technology, Ltd;
152. Shire Ag;
153. Sigilon;
154. Sigma Aldrich Co. Llc;
155. Sio2;
156. Ske S.R.L.;
157. Soil Culture Solutions Llc (Dba Soilcea);
158. Souffle Therapeutics
159. SQZ Biotechnologies;
160. StemBioSys, Inc.;
161. SuonoBio;
162. T2 Biosystems;
163. Taconic Biosciences, Inc. (formerly Taconic Farms);
164. Taiwania Capital (Bio-Asia Taiwan Symposium);
165. TARA;
166. Tarveda Therapeutics;
167. Teal Bio
168. Terasaki Institute
169. Tesio Pharmaceuticals
170. Third Rock Ventures;
171. Tiba Biotech LLC;
172. TissiuM (formerly"Gecko");
173. Transgenic Inc.;
174. Translate Bio (Formerly Rana Therapeutics, Inc.);
175. Trilink Biotechnologies, Inc.;
176. Unilever (Living Proof);
177. University of Bergen, Norway (Falch Lecture Honorarium);
178. VasoRX;
179. Verseau Therapeutics, Inc.;
180. Virex Health
181. Vitakey;
182. Vivtex Corporation;
183. Westlake University;
184. Whitehead Institute;
185. Wiki Foods;
186. Xenter;
187. YourBio (Formerly 7th Sense Biosystems)
188. Yz Biosciences (Guangzhou) Inc.;
189. Zenomics;
190. ZWI Therapeutics
